# High-depth, high-accuracy microsatellite genotyping enables precision lung cancer risk classification

**DOI:** 10.1038/onc.2017.256

**Published:** 2017-07-31

**Authors:** K R Velmurugan, R T Varghese, N C Fonville, H R Garner

**Affiliations:** 1 Department of Biological Sciences, Center for Bioinformatics and Genetics and the Primary Care Research Network, Edward Via College of Osteopathic Medicine, Blacksburg, VA, USA; 2 Department of Biological Sciences, Gibbs Cancer Center and Research Institute, Spartanburg, SC, USA; 3 Department of Biological Sciences, Riverside Law, LLP Glenhardie Corporate Center, Wayne, PA, USA

## Abstract

There remains a large discrepancy between the known genetic contributions to cancer and that which can be explained by genomic variants, both inherited and somatic. Recently, understudied repetitive DNA regions called microsatellites have been identified as genetic risk markers for a number of diseases including various cancers (breast, ovarian and brain). In this study, we demonstrate an integrated process for identifying and further evaluating microsatellite-based risk markers for lung cancer using data from the cancer genome atlas and the 1000 genomes project. Comparing whole-exome germline sequencing data from 488 TCGA lung cancer samples to germline exome data from 390 control samples from the 1000 genomes project, we identified 119 potentially informative microsatellite loci. These loci were found to be able to distinguish between cancer and control samples with sensitivity and specificity ratios over 0.8. Then these loci, supplemented with additional loci from other cancers and controls, were evaluated using a target enrichment kit and sample-multiplexed nextgen sequencing. Thirteen of the 119 risk markers were found to be informative in a well powered study (>0.99 for a 0.95 confidence interval) using high-depth (579x±315) nextgen sequencing of 30 lung cancer and 89 control samples, resulting in sensitivity and specificity ratios of 0.90 and 0.94, respectively. When 8 loci harvested from the bioinformatic analysis of other cancers are added to the classifier, then the sensitivity and specificity rise to 0.93 and 0.97, respectively. Analysis of the genes harboring these loci revealed two genes (*ARID1B* and *REL*) and two significantly enriched pathways (chromatin organization and cellular stress response) suggesting that the process of lung carcinogenesis is linked to chromatin remodeling, inflammation, and tumor microenvironment restructuring. We illustrate that high-depth sequencing enables a high-precision microsatellite-based risk classifier analysis approach. This microsatellite-based platform confirms the potential to create clinically actionable diagnostics for lung cancer.

## Introduction

Lung cancer has a high rate of incidence with 224 000 new cases projected this year alone: more than the next four cancers (colorectal, pancreatic, breast and prostate) combined.^1^ Only 18% of those diagnosed with lung cancer will survive 5 years; however, early detection can dramatically improve outcomes.^2^ About 80–85% of lung cancers are found to be non-small cell lung cancer (NSCLC).^3^ If found early the 5 year survival rate of NSCLC improves significantly: stage IA – 49%, stage IB – 54%, stage IIA – 30%, stage IIB – 31%, stage IIIA – 14%, stage IIIB – 5% and stage IV – 1%.^4^ The differing stage dependent survival rate and varying provenance of new lung cancers underscores the value of developing a lung cancer genetic risk diagnostic – especially for screening of ‘at risk’ populations (family members with lung cancer, second hand exposure to smoke or other hazards) which could be tested, with subsequent adjustments made to clinical observation or lifestyle. Interestingly, as the smoking rate has dropped in the US, it has been observed that ~20% of lung cancer deaths are from never smokers, attributable to other environmental exposures and genetic mutations.^5, 6^

Studies of disease specific variation have largely neglected repetitive DNA in favor of single nucleotide variants. However, an abundance of neurological disorders have been linked to length specific variations in repetitive DNA microsatellites (MST).^7^ These microsatellite loci consist of short (1–6 bp) units repeated in tandem. Recent studies have shown that microsatellites contribute to the genetic complexity of various cancers.^8, 9, 10^ Based on these previous findings it is hypothesized that microsatellites may play a role in the genetics of lung cancer.^11^

Our recent population-scale studies of MST loci and their repeat length variations have shown that MSTs can stratify risk, provide clinical decision support, and be potential therapeutic targets.^8, 9, 10, 12, 13, 14^ These observations were made possible by building robust computational pipelines to accurately genotype MST loci based repeat length variation.^8, 9, 10, 12^ Our previous work in computationally discovering clinically informative MST loci from publically available data sets (The Cancer Genome Atlas of affected individuals, the 1000 Genomes Project of healthy ‘normal’ individuals) have yielded disease specific germline MST loci variations for breast cancer, ovarian cancer, glioblastoma and lower-grade glioma and that these germline variants were rarely altered in matching tumors.^8, 9, 10, 12^ We have also shown somatic MST variability and the presence of minor alleles can act as indicative disease markers for colorectal and liver cancer.^15^ Furthermore, microsatellite variations are somatically acquired in normal tissues as one ages at rates higher than single nucleotide variants and that they are a sensitive measure of toxic environmental exposures.^16, 17^

The goal of this research was to discover and further evaluate a set of microsatellite markers for lung cancer risk via comparison of patient germline and normal control germline exome sequences. Marker validation utilizes a custom target enrichment kit for high-depth nextgen targeted sequencing. The focused, ultra-high read depth multiplexed sequencing approach used here enables accurate economical genotyping, validation and final selection of the most informative loci to ultimately create a high sensitivity and specificity risk classifier assay.

## Results

### Cancer risk classification pipeline

**Figure 1:**
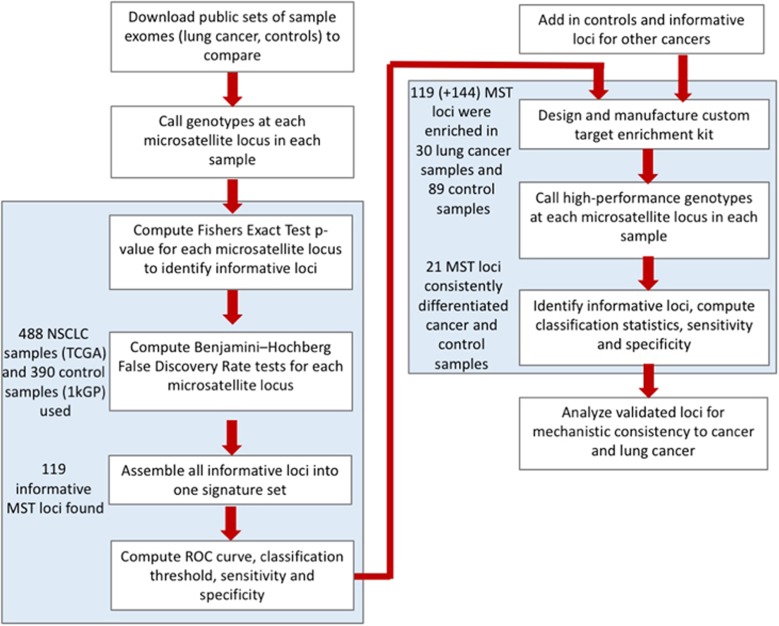
Flow chart of informative loci identification and marker validation. This work can be divided into two phases: the computational identification of informative MST loci phase and the marker validation phase. Phase 1: 488 non-small cell lung germline cancer samples from the TCGA and 390 germline non-cancer control samples from the 1000 genomes project we analyzed. This analysis yielded 119 MST loci that have significant genotype difference in the cancer and control samples. Phase 2: this set of 119 markers, along with 144 MST markers that were computationally found to be significant for others cancers, were pooled into a target enrichment kit which was used to sequence at high depth a total of 30 lung cancer samples and 89 non-cancer control samples. Of these 263 (119+144) MST markers, 21 were found to consistently differentiate lung cancer and control samples.

A computational pipeline was created for both candidate marker discovery and validation (Figure 1). The process to identify statistically informative MST loci and develop a classification signature (including receiver operating characteristic (ROC) curves and sensitivity and specificity calculations), follows the approach we have used previously for other cancers studies.^8, 9, 10, 12^ We applied part (depicted on the left side of Figure 1) of the pipeline to compute classifiers for lung adenocarcinoma (LUAD) germline samples vs normal germline controls and lung squamous cell carcinoma (LUSC) germline samples vs normal germline controls. Each of the individual locus found to be informative, that is, which passed statistical and false discovery tests were harvested for inclusion on a custom nextgen target enrichment kit. Those loci were supplemented with additional informative loci gathered from our previous reports of additional cancer types (breast, ovarian, melanoma and 3 different brain cancers) to identify potential pan-cancer markers. To the full set of informative loci were also added control loci that included random exon microsatellite loci, forensics/paternity testing loci and MSI (microsatellite stability) loci to verify performance of the enrichment kit.

Once a set of potentially informative microsatellite loci were identified (left side of Figure 1), lung cancer and control DNA samples were enriched for these markers using a custom specific microsatellite target enrichment kit (SMTEK) and sequenced at high depth with 16–48 samples multiplexed on each sequencing run (right side of Figure 1). The high-depth sequencing of these regions enabled calling of high-accuracy genotypes at each of the enriched loci. These genotypes were in turn used to further evaluate the consistency of those loci that could differentiate cancer from controls. ROC curves were computed for the informative loci from the lung cancer sets and for the lung cancer set plus informative loci from the other cancer types. Using these two verified sets of high-accuracy loci, we analyzed them for possible mechanistic (ontology, pathway, function, drug-ability and so on) relationships to illustrate their potential role in lung cancer.

### Analysis of whole-exome sequencing data for cancer and control germline samples

**Figure 2:**
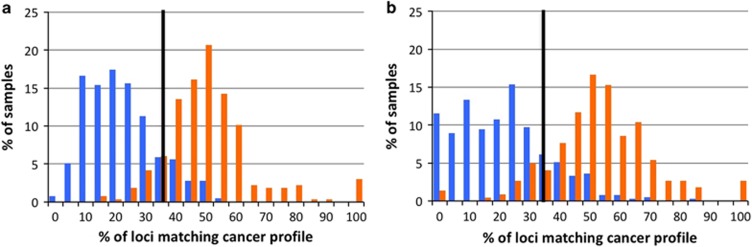
The computationally harvested LUAD and LUSC MST loci differentiate their corresponding cancer type from 1000 genomes non-cancer control samples with high sensitivity (LUAD: 0.87, LUSC: 0.88). (**a**) A sample with 39% (vertical black line; identified via ROC analysis) or more of the 96 LUAD specific MST loci with cancer genotype will be called ‘at-risk’ for adenocarcinoma of the lung. (**b**) A sample with 37% or more of the 67 LUSC specific MST loci with cancer genotype will be called ‘at-risk’ for squamous cell carcinoma. Blue bars represent control samples and orange bars represent lung cancer germline samples.

To compare MST genotypic variation in lung cancer germline samples and non-cancer germline control samples, 266 LUAD and 222 LUSC germline cancer exome samples were downloaded from The Cancer Genome Atlas (TCGA) and 390 germline non-cancer control exome sequencing data were downloaded from the 1000 Genomes Project (1kGP). With our 95% accurate^14^ MST allele calling method, on average, given the depth of the TCGA sequencing data, 50 thousand common microsatellite loci were analyzed by comparing modal (most frequent genotype in control samples) and non-modal genotype distributions in the two lung cancer sub-types and the non-cancer control samples. Two sets (one each for LUAD and LUSC) of MST loci were identified having significantly different genotypic distributions compared to non-cancer controls. Of these two sets, 96 LUAD and 67 LUSC MST loci (Supplementary Table 6) passed false discovery rate tests. A classification model that we previously developed to assess how well each set of markers differentiates the disease samples from healthy controls.^8, 9, 10^ The ROC demonstrates the predictive power of this classification scheme as well as the value of the underlying sets of loci: the area under the curve (AUC) is 0.94 (LUAD) and 0.92 (LUSC; Supplementary Figures 3 and 4). For each classification scheme an ‘at risk’ cutoff score was established by plotting accuracy vs cutoff, a sample with 39% or more of the 96 LUAD signature MST loci set with non-modal genotype will be classified as ‘at-risk’ for adenocarcinoma of the lung (Figure 2a) while a sample with 37% or more of the 67 LUSC signature MST loci set with non-modal genotype will be classified as ‘at-risk’ for squamous cell carcinoma (Figure 2b). The specificity and sensitivity of the LUAD classification scheme is 0.87 and 0.87, respectively. The specificity and sensitivity of the LUSC classification scheme is 0.82 and 0.88, respectively. The specificity and sensitivity of the classification power of the LUAD signature set was found to be 0.87 and 0.87; the same for the LUSC signature set was found to be 0.82 and 0.88.

### High-depth target sequencing of computationally harvested disease specific MST loci

To assess the differentiating power of the computationally harvested 119 (96 LUAD and 67 LUSC; of which 44 were in common) MST loci using high-depth enabled high-accuracy genotyping to further evaluate the computational findings, the 119 MST loci along with 144 MST loci computationally found to be specific for other cancers, and control loci were combined and enriched in 30 lung cancer samples (Supplementary Table 3) and 89 non-cancer control samples (Supplementary Table 4). Supplementary Figures 1 and 2 show that more than 93% of the loci were called in all the lung cancer and non-cancer control samples. The average read depth per loci across all samples was 579x (s.d., 315x). The minimum read depth was 83x.

### Identification of informative loci in the LUSC and LUAD MST loci sets

We successfully called the genotype for 105 out of 119 microsatellite markers that significantly differ in the lung cancer germline samples compared to non-cancer controls. Specifically, the predominant genotype for these 105 markers was calculated using high-depth sequencing of 30 lung cancer samples (Supplementary Table 3) and 89 non-cancer control samples (Supplementary Table 4). A subset of 13 markers (from the 105) were found to be informative, that is, having differing predominant genotypes between the high-depth lung cancer and non-cancer control data sets. The remaining non-informative loci were not included in the marker validation classifier computations.

### Genotyping MST loci from other diseases in the lung cancer samples

Recent findings from pan-cancer studies suggest that different cancer types share oncogenic signatures.^18, 19^ We investigated this possibility by including 144 informative MST loci identified in studies of breast cancer, ovarian cancer, lower-grade glioma, glioblastoma, melanoma and medulloblastoma to the target enrichment kit. Of the 144 loci, 137 loci were reliably genotyped in both sample groups. Among these, 8 loci (Table 1) were found to have differing predominant genotypes in the high-depth lung cancer and 1kGP non-cancer control data sets. The remainder of the loci were non-informative with respect to lung cancer.

### Performance of the high-depth informative loci as a classifier

**Figure 3:**
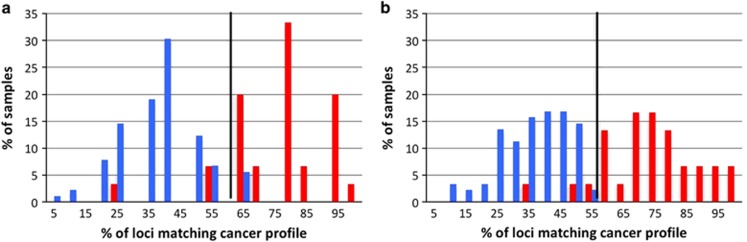
The 13 lung cancer specific MST loci and 8 MST loci specific for other diseases can differentiate between the lung cancer and non-cancer control sample groups. The blue and red bars represent the non-cancer control and lung cancer samples, respectively. (**a**) A sample with 61% or more of the 13 MST loci with cancer genotype will be termed ‘at-risk’ for lung cancer with sensitivity and specificity values of 0.90 and 0.94. (**b**) A sample with 57% or more of the 21 MST loci with cancer genotype will be termed ‘at-risk’ for lung cancer with sensitivity and specificity values of 0.93 and 0.97. The vertical black line corresponds to the optimum cutoff values found from the ROC analysis.

The same binary classification model employed to identify the candidate loci from the TCGA and 1000 Genomes data was used to assess the power of the 13 informative MST loci set (Table 1) to differentiate lung cancer samples from non-cancer control samples. The 13 MST loci signature differentiated lung cancer samples from non-cancer control samples with a sensitivity of 0.90 and specificity of 0.94. The area under the ROC curve was 0.96 (Supplementary Figure 5A). An optimal cutoff of 0.61 was identified by calculating the accuracy vs cutoff (Supplementary Figure 5B). This result has a simple interpretation: 8 or more predominant genotypes (out of 13) indicate an increased risk for NSCLC. (Figure 3a).

A similar classification model was computed for all 21 informative MST loci set (Table 1). This model has higher classification power with sensitivity and specificity values of 0.93 and 0.97, respectively. The area under the ROC curve is 0.97 (Supplementary Figure 6A). The accuracy vs cutoff plot suggests a cutoff of 0.57. The 21 MST classifier (Supplementary Figure 6B) shows that any sample with 57% or more of the 21 MST loci with predominant cancer genotype will be classified as ‘at-risk’ for NSCLC (Figure 3b).

The power for the marker validation experiment for both classification models was computed using an independent *t*-test for a sample size of 30 and a control size of 89, with a difference in population mean of 30 and a s.d. of 15. The power was >0.99 for a confidence interval of 0.95.^20^

Although the statistical power of the 21 MST loci to differentiate lung cancer from non-cancer control samples is significant, a leave one out cross validation was also performed to further quantify the performance of this model. The leave one out analysis (Methods section) predicted 28 out of 30 lung cancer samples to be ‘at-risk’ and 88 out of 89 non-cancer control samples to be ‘healthy’. The average sensitivity and specificity of this cross-validation effort, corresponding to the 119 leave one out iterations (due to the 30+89 sample count), was found to be 0.93 and 0.97, respectively (Supplementary Table 9 and Supplementary Figure 8). This cross validation demonstrates the consistency of this prediction method.

### Potential roles of the genes that harbor these informative loci

Of the 13 MST loci that are found to differentiate lung cancer samples from control samples, all were in the intronic regions of genes. To understand the potential mechanistic roles of these genes, the occurrence of mutations in these 13 genes were examined in 5 TCGA lung cancer studies. On average 37% of the lung cancer samples in these 5 studies contained mutations in at least one of the 13 genes (Supplementary Table 10). An LUSC study with 177 lung cancer samples had ~50% of the samples with mutations in at least one out of the 13 genes (Supplementary Table 10). Nine gene pairs were found to co-occur significantly (Supplementary Table 11). Of these gene pairs, the *REL* gene significantly co-occurred with 4 genes (*PPP1R21*, *CCDC88A*, *ATG3* and *PRPF18*) and *ARID1B* co-occurred with 2 genes (*IMPG1*, *FUBP3*). Interestingly, when these 13 genes were inspected for possible association with cancer using the COSMIC Cancer Gene Census,^21^ only *REL* and *ARID1B* were found to be previously implicated in cancer.^22, 23, 24^ When all 13 genes were examined for possible drug-ability, using DrugDB,^25^ *REL* and *ARID1B* were found to be clinically actionable. When clustering the 13 genes using the David ontology database we found alternative splicing (*P* value: 0.005) and splice variants (*P* value: 0.046) to be significant ontological characterizations (Supplementary Table 12). It should be noted that all the 13 MST loci that are found to be lung cancer differentiating are found in the intron regions of genes (Table 1). It has been shown previously that alterations in the MST loci in the intronic regions of the genes can influence transcription, alternative splicing or mRNA export to the cytoplasm.^26^ Upon further investigation of the 13 genes using Reactome,^27^ we found that two pathways were statistically enriched: the cellular response to stress pathway and the chromatin organization pathway.

## Discussion

Lung cancer is the leading cause of cancer-related mortality worldwide and early detection is vital to lessening the burden of this aggressive disease.^1, 2^ Our lab utilized available lung cancer and matched control germline nextgen sequences to identify risk markers that could be evaluated from a simple blood test. Those markers were incorporated in SMTEK, a next generation sequencing target enrichment kit, and its performance was verified on an independent set of samples and controls. This was made possible by extensively analyzing an understudied region of the genome, which has been found to be technically difficult to sequence^28^ and discovered a genomic signature of 13 loci which can be used to effectively predict cancer risk in lung cancer with high power, specificity and sensitivity.

Although substantial effort has been directed at identifying diagnostic actionable markers, there is a significant gap between the known genetic contributors to lung cancer and the number and power of known inherited and somatic variants.^29^ About 85% of all lung cancers are NSCLC, and with a better understanding of the heterogeneity of NSCLC, more patient specific treatment options are on the rise.^29^ The success of tailored lung cancer treatment arises from improvements in genetic and epigenetic biomarker discovery;^30^ however, early detection still remains the most significant factor in cancer survival. Important to early discovery is the identification of genetic risk markers, inherited or spontaneous, that will aid in identifying high-risk patients for enhanced monitoring or preventative measures. Recent studies have found that 20% of newly diagnosed lung cancer patients are never smokers, underscoring the need and potential for new genetic risk markers.^2, 5, 6^ The markers found in this study will potentially fill the gap by enabling risk stratification.

### High-depth MST genotyping

By calculating genotypes, on average, for 50 thousand MSTs in the two lung cancer sub-types in comparison with the non-cancer control samples, we found 67 LUSC and 96 LUAD MST loci (Supplementary Table 6) that can differentiate their corresponding lung cancer sub-type from the non-cancer controls at significant sensitivities and specificities (Supplementary Figures 3 and 4). Although we have previously demonstrated our genotyping accuracy from exome data sets to be 95%, the modest (~15x) read coverage in publically downloaded TCGA and 1000 Genome Project exome data sets limits the accuracy and ability to call genotypes at all loci.^13^ Low coverage, reduced sequence complexity and non-random variation, have hampered microsatellite-based biomarker discovery.^13, 30^ Although we have addressed these limitations in our previous efforts to identify cancer associated MST loci and tuned our genotyping algorithms accordingly, here we endeavored to mitigate the main source of genotyping error by dramatically increasing the depth of coverage at informative loci.^8, 9, 10, 12^ Hence, we specifically enriched 347 (disease and control) MST loci (Supplementary Table 1) in 119 multiplexed samples and attained an average per locus sample read depth of 579 (Supplementary Figure 2) which is ~20 times the usual exome read depth.^8, 9, 10, 12^ With high-depth enabled high-accuracy genotyping, 13 MST loci were found to have one predominant genotype (a genotype found in more than 50% of group members) that differed in the lung cancer and the non-cancer control groups (Supplementary Table 5). Eight MST loci previously found to be specific for other cancers were also able to differentiate lung cancer samples from the non-cancer control samples.

### 13 loci signature

The 13 MST loci were found to differentiate lung cancer samples from non-tumor control samples with sensitivity and specificity of 0.90 and 0.94 (Figure 3). All 13 loci were found in genes and the co-occurrence of these genes in the signature set is consistent with our mechanistic analyses. Mutation rates in these genes in five lung cancer TCGA studies show that 9 gene pairs were found to significantly co-occur (Supplementary Table 11). Of these 13 genes, *REL* and *ARID1B* have a previously established high mechanistic relevance to lung cancer. Mutations in *REL* was found to co-occur with mutations in 4 other signature genes (*PPP1R21*, *CCDC88A*, *ATG3* and *PRPF18*) and similarly *ARID1B* was found to co-occur with 2 signature genes (*IMPG1* and *FUBP3*; Supplementary Table 11). Hence the mutational co-occurrence of the signature genes in TCGA lung cancer studies is consistent with the 13 loci signature.

### Gene ontology

All 13 MST loci were found in the intron regions of their respective genes. Our previous findings show that a MST alteration in introns can influence the alternative splicing and gene transcription.^26^ Interestingly, when inspecting this cluster of 13 genes in the DAVID ontology database, we found alternative splicing (*P-*value: 0.005) and splice variants (*P*-value: 0.046) terms to be significantly enriched for these 13 genes (Supplementary Table 12).^31^

### Pathway analysis

**Figure 4:**
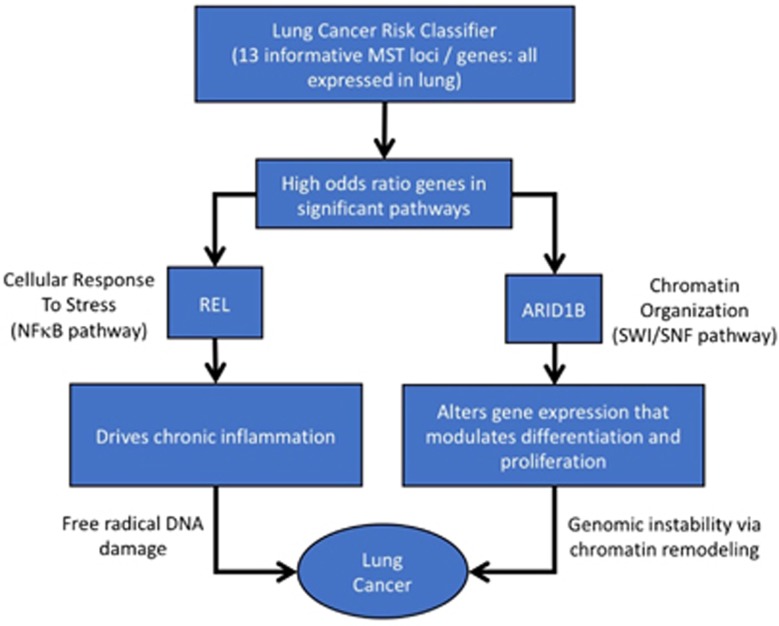
Schematic describing potential mechanism underlying lung carcinogenesis. Two genes out of 13 have significant oncogenic potential.

Interestingly, when these 13 genes were inspected for possible association with cancer using the COSMIC Cancer Gene Census, only *REL* and *ARID1B* were found to be previously implicated in cancer.^21^ TCGA analysis suggests *REL*, *ARID1B* and associated genes can drive lung carcinogenesis through DNA damage and chromatin remodeling induced genomic instability (Figure 4). Chromatin remodeling is a dynamic process which regulates DNA repair, recombination and gene transcription, which if impaired can play a pivotal role in carcinogenesis.^32, 33^ The cellular response to stress pathway is involved in damage control through protective or destructive cell response mechanisms that promote survival or initiate cell death.^34^

### Clinical action-ability: *REL* and *ARID1B*

We examined the drug-ability of these genes through DrugDB and found that currently two genes (*REL* and *ARID1B*) were categorized as clinically actionable.^25^ *ARID1B* is a transcriptional modulator of specific genes through chromatin remodeling. ARID1B is a part of the switch/sucrose non-fermenting (SWI/SNF) complex that is implicated in several cancers.^35^ More recently a study reported that loss of function in the SWI/SNF complex leads to genomic instability in lung cancer.^36^ Lung cancer sub-types showed no significant differences between histology, implying that the loss of SWI/SNF function caused genomic instability regardless of lung cancer sub-type, consistent with our observation that *ARID1B* is a marker for both LUAD and LUSC (Table 1).

*REL* is a proto-oncogene and member of the NFB transcription factor family. Rel/NFB transcription factors are critically involved in innate and adaptive immune responses through the up-regulation of chemokines, cytokines, cell adhesion molecules and proteases. The role of tobacco smoke as a carcinogen has been highly correlated with lung cancer and one explanation is the production of reactive oxygen species that is known to cause DNA damage and to activate NFB.^37^ It can be deduced that alterations in *REL* could predispose a smoker to increased risk of cancer compared to a non-smoker. Overexpression of *REL* has been associated to many lymphoid cancers such as primary mediastinal B-cell lymphoma, classical Hodgkin’s lymphoma, and solid tumors such as breast cancer, pancreatic cancer and head and neck cancer but not lung cancer.^22, 38, 39, 40, 41^ Interestingly, our lung cancer risk classifying locus harbored by the *REL* gene lies only 68 base pairs downstream of exon 6 and 17 base pairs upstream of exon 7, both of which are included in the *REL* homology domain. The *REL* homology domain is an N-terminal protein domain which is shared by *REL* genes, which mediates DNA binding, inhibitor binding, nuclear localization signal and dimerization.^37^ It can be inferred that intronic mutations located in between two exons in close proximity of each other can affect protein structure in the *REL* homology domain that can influence downstream effects of the NFB pathway and consequently predispose individuals to cancer. Further research is needed to understand the mutational impact of this loci in terms of *REL* localization, effect on the NFB pathway, and its role in lung tumorigenesis.

## Conclusion

Taken together, our results propose a 13 loci lung cancer risk classifier that may reveal insight into the mechanism of lung carcinogenesis. Dysfunctions in the two significantly enriched pathways can possibly encourage lung carcinogenesis through chromatin remodeling, inflammation and tumor microenvironment restructuring. The genes *ARID1B* and *REL* are of special interest because of their drug availability, oncogenic implications, odds risk ratio scores (Supplementary Table 11) and co-occurrence with other implicated loci. These findings may be of interest because of the clinical potential value of this lung cancer risk classifier for novel therapeutic target discovery, lung cancer prediction and cancer risk assessment.

## Methods

### Computational identification of LUAD and LUSC specific MST loci

A total of 266 LUAD and 222 LUSC germline exome samples were downloaded from TCGA. For the non-tumor control population, 390 germline exome samples were downloaded from the 1kGP. The LUSC and LUAD samples are ethnically matched with the 1kGP non-tumor control samples and the gender, age, smoking status and ethnicity of these samples are as given in Supplementary Table 13 and Supplementary Figure 7. On average, a set of 50 thousand MST loci out of a maximum of 1.8 million, that were extracted from the human genome (38 build) using Tandem Repeat Finder, could be genotyped in these samples, set by the sequencing depth of the samples. A modal genotype was computed for each MST locus using the 1kGP samples. A 2X2 Fisher’s exact test was computed for each locus comparing the modal and non-modal genotype distributions in these two samples groups.^10^ A Benjamini-Hochberg cutoff of 0.01% was used as a false discovery rate cutoff. A binary classifier was generated using ROCR library in R for the two MST loci lists to determine their potential to differentiate their corresponding lung cancer sub-type from the normal control samples.^42, 43^

### Assembling informative MST loci set for target enrichment

A set of 347 loci was assembled into the Illumina TruSeq Amplicon V1.5 kit target enrichment kit (Supplementary Table 1). Of the 347, 119 were found to be specific for lung cancer, 144 were found in similar manner by analyzing other cancer data sets (Supplementary Table 2) and 84 were included as controls (Supplementary Tables 6–8).

### Genomic DNA library prep and sequencing

Thirty lung cancer samples and 89 B-Lymphocyte non-tumor samples were obtained from Origene and Coriell cell repositories. The cells were cultured following the suppliers’ recommended conditions (Supplementary Tables 3 and 4). Isolation of genomic DNA was done using the Qiagen DNA Blood and Tissue kit following the manufacturer’s protocol. Our previously published studies in breast and ovarian cancer and in glioblastoma show that MST genotype in matched cancer tissue samples and germline samples from cancer patients do not vary to a larger extent and explains our usage of cancer tissue DNA samples for the marker validation set.^8, 9, 10^

### Target sequencing, SMTEK

The assembled set of 347 MST loci was uploaded to Illumina’s Design Studio tool, obtained in the form of a target enrichment kit and was used to target enrich and sequence the 30 lung cancer samples and 89 control samples.

### Genotyping of target enriched samples

The 347 MST loci were genotyped in the target enriched samples using custom written scripts, after performing quality control steps using the Trimmomatic tool to ensure only high quality reads are used in genotyping.^44^ For each locus, a modal genotype and a predominant cancer genotype was computed. A modal genotype is the genotype that is found in more than 50% of the control samples and the predominant cancer genotype is the genotype that is found in more than 50% of the lung cancer samples. Any locus that has differing modal genotype and predominant cancer genotype was considered as a risk classifier (Supplementary Table 5).

### Statistical procedure to assess differentiating power of the informative risk markers

Of the 119 computationally found lung cancer specific MST loci, 13 were found to differentiate lung cancer and normal control marker validation samples. A binary classifier was generated using the ROCR library in R using the 13 MST loci to assess their statistical capacity to separate lung cancer samples from normal control samples. These 13 MST loci represent both the LUAD and LUSC samples, for a combined lung cancer risk computation that was sufficiently powered in the target enrichment experiment. The sensitivity, specificity and other ROC related calculations were computed using the ROCR library in R. Odds ratio was calculated using the formula: (TP/FP)/(FN/TN), where TP, FP, FN and TN are true positive, false positive, false negative and true negative, respectively.^45^ A set of 8 MST loci that were computationally found to be specific for other cancers were also found to differentiate the lung cancer samples from the normal control samples. This set was added to the 13 MST loci to form a 21 MST set. A similar statistical assessment was performed with this loci set. A leave one out cross validation was performed to quantify the consistency of the predictive power of the 21 loci classifier.

### Mechanistic analysis

Genes for each marker were identified from the UCSC genome browser referencing HG38. Functional enrichment analysis of genes harboring microsatellite markers and gene ontologies were obtained through the David Bioinformatics 6.8 Database.^46^ Pathway analyses were performed using the Reactome database.^27^ Alterations and co-occurrence/mutual exclusivity of genes in gene set were analyzed in TCGA lung cancer studies using cbioportal.^31^ Studies included in cbioportal analyses were: LUAD,^47^ LUAD (TCGA, Provisional), LUSC,^48^ LUSC (TCGA, Provisional), and Pan-Lung Cancer.^49^ Drug-ability of gene set was analyzed using the DGIdb database.^25^

## Code availability

Computer code used to genotype MSTs from the high-depth sequencing data for the marker validation data sets can be requested from the corresponding author through email.

## Figures and Tables

**Table 1:** MST loci that can precisely differentiate between the lung cancer samples and non-tumor samples

*Genomic position*	*Repeat*	*Gene region*	*Gene*	*Entrez ID*	*Disease*	*Odds ratio*
chr2:60918364-60918376	T	Intron	*REL*	5966	LUAD, LUSC	39.92
chr6:157174818-157174831	T	Intron	*ARID1B*	57 492	LUAD, LUSC, MB, SKCM	13.57
chr6:76018867-76018880	A	Intron	*IMPG1*	3617	LUSC, OV	12.28
chr3:94035443-94035458	T	Intron	*ARL13B*	200 894	GBM, LUAD, SKCM	11.20
chr3:112534347-112534360	A	Intron	*ATG3*	64 422	GBM, LGG, LUAD, LUSC	10.29
chr8:129862369-129862381	A	Intron	*FAM49B*	51 571	LUAD, LUSC	7.01
chr9:130622843-130622857	A	Intron	*FUBP3*	8939	GBM, LGG, LUAD, LUSC, MB, OV	6.93
chr7:135414296-135414309	A	Intron	*CNOT4*	4850	LUAD	5.07
chr2:48461120-48461133	T	Intron	*KLRAQ1*	129 285	LGG, LUAD, LUSC, MB, SKCM	4.43
chr2:55332516-55332530	A	Intron	*CCDC88A*	55 704	LUAD, LUSC, SKCM	3.90
chr13:31148484-31148500	A	Intron	*HSPH1*	3315	LUAD, SKCM	3.70
chr15:20458509-20458521	A	Intron	*HERC2P3*	283 755	LUAD	3.00
chr10:13591929-13591943	T	Intron	*PRPF18*	8559	LUSC	2.25
chr2:202815832-202815844	A	Intron	*ICA1L*	130 026	BC, GBM, OV	7.61
chr13:114236623-114236635	T	Intron	*CDC16*	8881	LGG, SKCM	6.19
chr12:106106383-106106396	A	Intron	*NUAK1*	9891	OV	5.74
chr3:98580864-98580876	A	Intron	*CPOX*	1371	BC, OV	5.02
chr16:70839964-70839978	T	Intron	*HYDIN*	54 768	GBM	4.58
chr2:233460070-233460083	A	Intron	*DGKD*	8527	OV	3.82
chr5:87383860-87383873	T	Intron	*RASA1*	5921	OV	2.93
chr8:23852057-23852082	TG	Intron	*STC1*	6781	BC	1.87

Abbreviations: GBM, glioblastoma; LGG, lower grade glioma; LUAD, lung adenocarcinoma; LUSC, lung squamous cell carcinoma. MB, medulloblastoma; OV, ovarian cancer; SKCM, skin cutaneous melanoma.
