# Supplementary material for: High-depth, high-accuracy microsatellite genotyping enables precision lung cancer risk classification
Source: Oncogene. 2017 Jul 31;36(46):6383–90. doi: 10.1038/onc.2017.256 (PMC5701090; doi:10.1038/onc.2017.256)
Supplement: Supplementary Material [file onc2017256x1.docx]

**Supplementary table 1: The Specific Microsatellite Target Enrichment Kit (SMTEK) consisted of 347 MST loci of which 322 were genotyped in at least 10 tumor and non-cancer control samples.** All 322 successfully genotyped loci had at least 10 reads in all the samples they were called.

| **Loci Type** | **Total before sequencing** | **After sequencing** | | |
| --- | --- | --- | --- | --- |
|  |  | **Passed** | **Failed** | |
| Disease | 263 | 242 | 21 |  |
| Control | 84 | 79 | 5 |  |
| Total | 347 | 322 | 28 |  |

**Supplementary table 2:** **Genotyping all MST loci in several TCGA germline cancer sample types and comparing them to 1000 Genome Project sample genotypes yield disease specific sets of MSTs that can differentiate cancer and non-cancer control samples.** Note, some loci were found to be informative for several cancer disease types

| **Cancer disease type** | **# MST loci** |
| --- | --- |
| Lung squamous cell carcinoma | 67 |
| Lung adenocarcinoma | 96 |
| Skin cutaneous melanoma | 68 |
| Breast cancer | 55 |
| Ovarian cancer | 57 |
| Glioblastoma | 48 |
| Medulloblastoma | 25 |
| Lower grade glioma | 38 |

Lung squamous cell carcinoma = LUSC; Lung adenocarcinoma = LUAD; Skin cutaneous melanoma = SKCM; Breast cancer = BC; Ovarian cancer = OV; Glioblastoma = GBM; Medulloblastoma = MB; Lower grade glioma = LGG.

**Supplementary table 3:** Details of the 30 lung cancer samples used to validate the LUSC and LUAD markers via SMTEK nextgen sequencing. All these samples are of Caucasian ethnicity. The age at diagnosis for these samples ranged between 41 and 84. The smoking status of the lung cancer samples were not available. These samples are ethnically matched with the 1000 genome samples used as controls. It should be noted that lung cancer tissue DNA samples are used in place of lung cancer germline DNA samples for the high-depth validation. Three of our previously published studies that compare MST genotype between matched cancer tissue DNA samples and germline DNA samples from cancer patients in breast, ovarian and brain cancer show that these two sample groups have the same MST genotype, and that the classification using tumor derived DNA is an accurate surrogate for germline DNA^1-3^.

| **Sample #** | **Tissue** | **Sample source** |
| --- | --- | --- |
| 1 | Lung cancer | Origene 3398 |
| 2 | Lung cancer | Origene 3538 |
| 3 | Lung cancer | Origene 3619 |
| 4 | Lung cancer | Origene 3803 |
| 5 | Lung cancer | Origene 3842 |
| 6 | Lung cancer | Origene 3844 |
| 7 | Lung cancer | Origene 3863 |
| 8 | Lung cancer | Origene 3931 |
| 9 | Lung cancer | Origene 3950 |
| 10 | Lung cancer | Origene 3989 |
| 11 | Lung cancer | Origene 4031 |
| 12 | Lung cancer | Origene 4033 |
| 13 | Lung cancer | Origene 4056 |
| 14 | Lung cancer | Origene 4119 |
| 15 | Lung cancer | Origene 4176 |
| 16 | Lung cancer | Origene CD07 |
| 17 | Lung cancer | Origene CD22 |
| 18 | Lung cancer | Origene CD26 |
| 19 | Lung cancer | Origene CD28 |
| 20 | Lung cancer | Origene CD36 |
| 21 | Lung cancer | Origene CD44 |
| 22 | Lung cancer | Origene CD56 |
| 23 | Lung cancer | Origene CD57 |
| 24 | Lung cancer | Origene CD59 |
| 25 | Lung cancer | Origene CD61 |
| 26 | Lung cancer | Origene CD62 |
| 27 | Lung cancer | Origene CD66 |
| 28 | Lung cancer | Origene CD71 |
| 29 | Lung cancer | Origene CD77 |
| 30 | Lung cancer | Origene CD97 |

**Supplementary table 4:** **Details of the 1000 genomes germline samples used as control samples, i.e. were normal healthy individuals. All these samples are of Caucasian ethnicity.** The age information of these samples was not available. The 1000 genomes samples are from healthy individual and hence non-smokers. These samples are from British and Finnish population and hence are ethnically matched with the lung cancer sample group.

| **Sample #** | **Cell type** | **Sample source** |
| --- | --- | --- |
| 1 | B-Lymphocyte | Coriell HG00313 |
| 2 | B-Lymphocyte | Coriell HG00180 |
| 3 | B-Lymphocyte | Coriell HG00181 |
| 4 | B-Lymphocyte | Coriell HG00138 |
| 5 | B-Lymphocyte | Coriell HG00113 |
| 6 | B-Lymphocyte | Coriell HG00100 |
| 7 | B-Lymphocyte | Coriell HG00116 |
| 8 | B-Lymphocyte | Coriell HG00107 |
| 9 | B-Lymphocyte | Coriell HG00278 |
| 10 | B-Lymphocyte | Coriell HG00269 |
| 11 | B-Lymphocyte | Coriell HG00125 |
| 12 | B-Lymphocyte | Coriell HG00182 |
| 13 | B-Lymphocyte | Coriell HG00272 |
| 14 | B-Lymphocyte | Coriell HG00273 |
| 15 | B-Lymphocyte | Coriell HG00121 |
| 16 | B-Lymphocyte | Coriell HG00186 |
| 17 | B-Lymphocyte | Coriell HG00134 |
| 18 | B-Lymphocyte | Coriell HG00131 |
| 19 | B-Lymphocyte | Coriell HG00190 |
| 20 | B-Lymphocyte | Coriell HG00102 |
| 21 | B-Lymphocyte | Coriell HG00127 |
| 22 | B-Lymphocyte | Coriell HG00135 |
| 23 | B-Lymphocyte | Coriell HG00110 |
| 24 | B-Lymphocyte | Coriell HG00274 |
| 25 | B-Lymphocyte | Coriell HG00118 |
| 26 | B-Lymphocyte | Coriell HG00115 |
| 27 | B-Lymphocyte | Coriell HG00187 |
| 28 | B-Lymphocyte | Coriell HG00104 |
| 29 | B-Lymphocyte | Coriell HG00111 |
| 30 | B-Lymphocyte | Coriell HG00122 |
| 31 | B-Lymphocyte | Coriell HG00277 |
| 32 | B-Lymphocyte | Coriell HG00139 |
| 33 | B-Lymphocyte | Coriell HG00282 |
| 34 | B-Lymphocyte | Coriell HG00183 |
| 35 | B-Lymphocyte | Coriell HG00309 |
| 36 | B-Lymphocyte | Coriell HG00119 |
| 37 | B-Lymphocyte | Coriell HG00268 |
| 38 | B-Lymphocyte | Coriell HG00310 |
| 39 | B-Lymphocyte | Coriell HG00097 |
| 40 | B-Lymphocyte | Coriell HG00312 |
| 41 | B-Lymphocyte | Coriell HG00108 |
| 42 | B-Lymphocyte | Coriell HG00308 |
| 43 | B-Lymphocyte | Coriell HG00178 |
| 44 | B-Lymphocyte | Coriell HG00132 |
| 45 | B-Lymphocyte | Coriell HG00266 |
| 46 | B-Lymphocyte | Coriell HG00129 |
| 47 | B-Lymphocyte | Coriell HG00117 |
| 48 | B-Lymphocyte | Coriell HG00099 |
| 49 | B-Lymphocyte | Coriell HG00136 |
| 50 | B-Lymphocyte | Coriell HG00133 |
| 51 | B-Lymphocyte | Coriell HG00171 |
| 52 | B-Lymphocyte | Coriell HG00188 |
| 53 | B-Lymphocyte | Coriell HG00275 |
| 54 | B-Lymphocyte | Coriell HG00176 |
| 55 | B-Lymphocyte | Coriell HG00306 |
| 56 | B-Lymphocyte | Coriell HG00103 |
| 57 | B-Lymphocyte | Coriell HG00140 |
| 58 | B-Lymphocyte | Coriell HG00098 |
| 59 | B-Lymphocyte | Coriell HG00281 |
| 60 | B-Lymphocyte | Coriell HG00177 |
| 61 | B-Lymphocyte | Coriell HG00109 |
| 62 | B-Lymphocyte | Coriell HG00271 |
| 63 | B-Lymphocyte | Coriell HG00106 |
| 64 | B-Lymphocyte | Coriell HG00105 |
| 65 | B-Lymphocyte | Coriell HG00137 |
| 66 | B-Lymphocyte | Coriell HG00128 |
| 67 | B-Lymphocyte | Coriell HG00124 |
| 68 | B-Lymphocyte | Coriell HG00096 |
| 69 | B-Lymphocyte | Coriell HG00142 |
| 70 | B-Lymphocyte | Coriell HG00284 |
| 71 | B-Lymphocyte | Coriell HG00120 |
| 72 | B-Lymphocyte | Coriell HG00285 |
| 73 | B-Lymphocyte | Coriell HG00276 |
| 74 | B-Lymphocyte | Coriell HG00123 |
| 75 | B-Lymphocyte | Coriell HG00173 |
| 76 | B-Lymphocyte | Coriell HG00280 |
| 77 | B-Lymphocyte | Coriell HG00112 |
| 78 | B-Lymphocyte | Coriell HG00174 |
| 79 | B-Lymphocyte | Coriell HG00101 |
| 80 | B-Lymphocyte | Coriell HG00311 |
| 81 | B-Lymphocyte | Coriell HG00179 |
| 82 | B-Lymphocyte | Coriell HG00114 |
| 83 | B-Lymphocyte | Coriell HG00267 |
| 84 | B-Lymphocyte | Coriell HG00130 |
| 85 | B-Lymphocyte | Coriell HG00126 |
| 86 | B-Lymphocyte | Coriell HG00189 |
| 87 | B-Lymphocyte | Coriell HG00141 |
| 88 | B-Lymphocyte | Coriell HG00270 |
| 89 | B-Lymphocyte | Coriell HG00185 |

**Supplementary table 5: A set of 13 lung cancer specific loci and 8 other loci that were found to be specific for other diseases were found to have differing genotypes in both the sample groups.** All the 21 loci have predominant genotypes (larger than the sum of all the other genotypes, i.e. more than 50%) in both the groups. The genomic coordinates furnished correspond to the HG38 genome reference build.

| **Genomic position** | **PD control GT** | **PD control GT sample %** | **PD cancer GT** | **PD cancer GT sample %** | **Odds ratio** |
| --- | --- | --- | --- | --- | --- |
| chr2:60918364-60918376 | 13_13 | 49/89 (55%) | 13_12 | 29/30 (97%) | 39.92 |
| chr6:157174818-157174831 | 14_14 | 51/89 (57%) | 14_13 | 27/30 (90%) | 13.57 |
| chr6:76018867-76018880 | 14_14 | 60/85 (71%) | 14_13 | 24/30 (80%) | 12.28 |
| chr3:94035443-94035458 | 16_16 | 46/88 (52%) | 16_15 | 27/30 (90%) | 11.20 |
| chr3:112534347-112534360 | 15_15 | 46/83 (55%) | 15_14 | 26/30 (87%) | 10.29 |
| chr8:129862369-129862381 | 13_13 | 57/86 (66%) | 13_12 | 22/30 (73%) | 7.01 |
| chr9:130622843-130622857 | 15_15 | 55/84 (65%) | 15_14 | 22/30 (73%) | 6.93 |
| chr7:135414296-135414309 | 14_14 | 47/87 (54%) | 14_13 | 23/30 (77%) | 5.07 |
| chr2:48461120-48461133 | 14_14 | 69/89 (78%) | 14_13 | 16/30 (53%) | 4.43 |
| chr2:55332516-55332530 | 15_15 | 53/85 (62%) | 15_14 | 20/30 (67%) | 3.90 |
| chr13:31148484-31148500 | 17_16 | 43/83 (52%) | 16_15 | 18/30 (60%) | 3.70 |
| chr15:20458509-20458521 | 13_12 | 48/89 (54%) | 12_11 | 20/30 (67%) | 3.00 |
| chr10:13591929-13591943 | 15_15 | 56/89 (63%) | 15_14 | 16/30 (53%) | 2.25 |
| chr2:202815832-202815844 | 13_13 | 69/85 (81%) | 13_12 | 18/30 (60%) | 7.61 |
| chr13:114236623-114236635 | 13_13 | 51/89 (57%) | 13_12 | 24/30 (80%) | 6.19 |
| chr12:106106383-106106396 | 14_14 | 40/61 (66%) | 14_13 | 14/22 (64%) | 5.74 |
| chr3:98580864-98580876 | 13_13 | 50/83 (60%) | 13_12 | 22/30 (73%) | 5.02 |
| chr16:70839964-70839978 | 15_12 | 59/89 (66%) | 12_12 | 19/30 (63%) | 4.58 |
| chr2:233460070-233460083 | 14_14 | 44/88 (50%) | 14_13 | 23/30 (77%) | 3.82 |
| chr5:87383860-87383873 | 14_14 | 45/83 (54%) | 14_13 | 20/30 (67%) | 2.93 |
| chr8:23852057-23852082 | 26_26 | 44/84 (52%) | 26_24 | 16/30 (53%) | 1.87 |

The classifier: After obtaining the list of markers that can differentiate between lung cancer samples and control samples, the classifier is built as follows: 1. For each sample, both cancer and control, all the MSTs in the list are genotyped. 2. The percentage of MSTs with the same genotype as the cancer genotype is calculated. 3. The binary classification value is assigned to each sample. The cancer samples are assigned the value “1” and the control samples are assigned the value “0”. 4. A table is generated with three values: the sample name, the fraction (%) of callable MST loci list with cancer genotype and the binary classification value. 5. This table is fed to the ROCR library in R which produces three output values and two graphs. The output values are sensitivity, specificity and cutoff. The sensitivity and specificity values shows the ability of the MST list in differentiating cancer and control samples. The cutoff value gives the cutoff % (percentage of list-MSTs with cancer genotype in a sample) beyond which a sample can be called “cancer-like”. The two graphs that are produced are the ROC plot and the accuracy vs. cutoff plot.

PD – predominant.

**Supplementary table 6:**  **Out of 119 MST loci computationally found to be specific for LUAD and/or LUSC cancer types, 105 produced data in both sample groups.** All loci produced at least 10 reads per loci in at least 10 samples in both sample groups. The genomic coordinates furnished attribute to the HG38 build.

| **Genomic position** | **Repeat** | **Gene region** | **Gene** | **Disease** |
| --- | --- | --- | --- | --- |
| chr6:36484827-36484842 | A | intron | KCTD20 | BC, GBM, LGG, LUAD, LUSC, SKCM |
| chr13:44943348-44943377 | AC | intron | NUFIP1 | BC, GBM, LUAD |
| chrX:13757634-13757649 | T | intron | OFD1 | BC, GBM, LUAD |
| chr1:10297149-10297165 | T | intron | KIF1B | BC, LGG, LUAD, SKCM |
| chr17:16070104-16070120 | T | intron | NCOR1 | BC, LGG, LUSC, SKCM |
| chr14:50881564-50881580 | T | intron | ABHD12B | BC, LUSC |
| chr3:112534347-112534360 | A | intron | ATG3 | GBM, LGG, LUAD, LUSC |
| chr9:130622843-130622857 | A | intron | FUBP3 | GBM, LGG, LUAD, LUSC, MB, OV |
| chr17:42834438-42834469 | GA | intron | PSME3 | GBM, LGG, LUAD, LUSC, MB, SKCM |
| chr13:113310584-113310595 | T | intron | LAMP1 | GBM, LGG, LUAD, LUSC, MB, SKCM |
| chr4:5745180-5745201 | TTC | intron | EVC | GBM, LGG, LUAD, LUSC, MB, SKCM |
| chrX:132097403-132097440 | AC | intron | FRMD7 | GBM, LUAD |
| chr10:33182834-33182862 | CA | intron | NRP1 | GBM, LUAD |
| chr6:31864580-31864594 | A | intron | SLC44A4 | GBM, LUAD, LUSC |
| chr2:86894983-86894997 | T | intergenic | - | GBM, LUAD, LUSC, MB |
| chr1:16564320-16564331 | A | intron | NBPF1 | GBM, LUAD, LUSC, MB |
| chr15:84512873-84512887 | A | 3utr | FLJ40113 | GBM, LUAD, MB |
| chr1:111762785-111762800 | A | intron | DDX20 | GBM, LUAD, SKCM |
| chr3:94035443-94035458 | T | intron | ARL13B | GBM, LUAD, SKCM |
| chr3:196361939-196361954 | A | intron | UBXN7 | LGG, LUAD |
| chr4:127699990-127700002 | T | intron | INTU | LGG, LUAD |
| chr3:50118451-50118476 | GA | exon | RBM5 | LGG, LUAD, LUSC |
| chr2:110963566-110963604 | TG | intron | ACOXL | LGG, LUAD, LUSC, MB, SKCM |
| chr13:27559820-27559834 | A | intron | LNX2 | LGG, LUAD, LUSC, MB, SKCM |
| chr7:65961068-65961081 | A | intron | GUSB | LGG, LUAD, LUSC, MB, SKCM |
| chr2:48461120-48461133 | T | intron | KLRAQ1 | LGG, LUAD, LUSC, MB, SKCM |
| chr16:66912992-66913023 | GT | intron | CDH16 | LGG, LUAD, LUSC, SKCM |
| chr12:95094564-95094577 | A | intron | FGD6 | LGG, LUAD, LUSC, SKCM |
| chr4:112186674-112186688 | T | intron | C4orf32 | LGG, LUAD, LUSC, SKCM |
| chr13:77217965-77217977 | A | intron | MYCBP2 | LGG, LUAD, LUSC, SKCM |
| chr15:73126401-73126414 | T | intron | NEO1 | LGG, LUAD, LUSC, SKCM |
| chr5:137677662-137677675 | A | intron | KLHL3 | LGG, LUAD, LUSC, SKCM |
| chr15:43710473-43710501 | TG | intergenic | - | LGG, LUAD, LUSC, SKCM |
| chr5:72889765-72889779 | T | intron | TNPO1 | LGG, LUAD, LUSC, SKCM |
| chr4:22442629-22442643 | A | intron | GPR125 | LGG, LUAD, OV, SKCM |
| chr9:115402097-115402108 | T | intron | DEC1' | LGG, LUAD, SKCM |
| chr21:43068646-43068659 | A | intron | CBS | LGG, LUSC |
| chr8:7359489-7359500 | T | intergenic | - | LUAD |
| chr3:46709584-46709610 | AAG | exon | TMIE | LUAD |
| chr16:19608281-19608294 | T | intron | C16orf62 | LUAD |
| chr12:80845943-80845957 | A | intron | LIN7A | LUAD |
| chr12:118383996-118384007 | T | intron | SUDS3 | LUAD |
| chrX:133216975-133216989 | A | exon | TFDP3 | LUAD |
| chr4:84635240-84635255 | T | intron | CDS1 | LUAD |
| chr14:24102652-24102664 | A | intron | PCK2 | LUAD |
| chr14:58208106-58208119 | A | intron | ACTR10 | LUAD |
| chr14:62950568-62950583 | A | intron | KCNH5 | LUAD |
| chr7:135414296-135414309 | A | intron | CNOT4 | LUAD |
| chr6:13316549-13316562 | A | intron | TBC1D7 | LUAD |
| chr6:95586994-95587006 | TA | intron | MANEA | LUAD |
| chr15:43635426-43635437 | A | intron | CATSPER2 | LUAD |
| chr15:20458509-20458521 | A | intergenic | - | LUAD |
| chr1:186361564-186361576 | A | intron | TPR | LUAD |
| chr1:1988996-1989009 | A | intron | KIAA1751 | LUAD |
| chr18:74129655-74129666 | A | intron | FBXO15 | LUAD |
| chr5:172994758-172994772 | T | intron | ATP6V0E1 | LUAD |
| chr5:157098910-157098931 | AG | intron | HAVCR2 | LUAD |
| chr6:7595009-7595021 | T | intron | SNRNP48 | LUAD |
| chr2:190673068-190673082 | T | intron | NAB1 | LUAD |
| chr3:108505839-108505853 | A | intron | MYH15 | LUAD |
| chr3:132502233-132502248 | T | intron | DNAJC13 | LUAD |
| chr11:89914202-89914215 | A | intron | LOC729384 | LUAD |
| chr16:74893610-74893622 | A | intron | WDR59 | LUAD |
| chr2:60918364-60918376 | T | intron | REL | LUAD, LUSC |
| chr6:157901855-157901868 | T | intron | SNX9 | LUAD, LUSC |
| chr16:12051827-12051841 | T | upstream | SNX29 | LUAD, LUSC |
| chr12:96912826-96912839 | T | 5utr | NEDD1 | LUAD, LUSC |
| chr14:81108485-81108507 | T | intron | TSHR | LUAD, LUSC |
| chr4:109347460-109347474 | T | intergenic | - | LUAD, LUSC |
| chr4:44689779-44689792 | A | intron | GUF1 | LUAD, LUSC |
| chr10:104038216-104038281 | AC | intron | COL17A1 | LUAD, LUSC |
| chr20:33367014-33367027 | A | intron | CDK5RAP1 | LUAD, LUSC |
| chr8:129862369-129862381 | A | intron | FAM49B | LUAD, LUSC |
| chr8:109523216-109523230 | T | intron | PKHD1L1 | LUAD, LUSC |
| chr11:108188043-108188057 | T | intron | NPAT | LUAD, LUSC |
| chr11:124754810-124754824 | A | intron | ESAM | LUAD, LUSC |
| chr3:161238095-161238109 | T | intron | NMD3 | LUAD, LUSC, MB, SKCM |
| chr6:157174818-157174831 | T | intron | ARID1B | LUAD, LUSC, MB, SKCM |
| chr11:108271229-108271243 | T | intron | ATM | LUAD, LUSC, MB, SKCM |
| chr4:185267220-185267233 | A | intron | SNX25 | LUAD, LUSC, OV |
| chrX:52753170-52753183 | T | intron | SSX2 | LUAD, LUSC, SKCM |
| chr6:136389532-136389546 | A | intron | MAP7 | LUAD, LUSC, SKCM |
| chr22:35323386-35323400 | A | intron | TOM1 | LUAD, LUSC, SKCM |
| chr2:55332516-55332530 | A | intron | CCDC88A | LUAD, LUSC, SKCM |
| chr1:62578975-62578989 | A | intron | DOCK7 | LUAD, LUSC, SKCM |
| chr4:73144839-73144853 | A | intron | ANKRD17 | LUAD, MB |
| chrX:52895580-52895606 (HG19) | GT | intron | XAGE3 | LUAD, MB |
| chr16:10689232-10689244 | A | intron | TEKT5 | LUAD, OV |
| chr2:24327999-24328012 | A | intron | ITSN2 | LUAD, SKCM |
| chr13:31148484-31148500 | A | intron | HSPH1 | LUAD, SKCM |
| chr7:5199689-5199704 | A | intron | WIPI2 | LUAD, SKCM |
| chr1:172608725-172608738 | T | intron | C1orf9 | LUAD, SKCM |
| chr1:100077147-100077161 | T | intron | HIAT1 | LUAD, SKCM |
| chr4:165467674-165467685 | T | intron | CPE | LUAD, SKCM |
| chrX:48354751-48354764 | A | intron | SSX3 | LUAD, SKCM |
| chr12:21638477-21638491 | A | intron | LDHB | LUSC |
| chr10:13591929-13591943 | T | intron | PRPF18 | LUSC |
| chr3:186804505-186804518 | A | intron | RFC4 | LUSC |
| chr19:6833152-6833167 | T | intron | VAV1 | LUSC |
| chr1:153645035-153645049 | T | intron | C1orf77 | LUSC, MB, SKCM |
| chr6:76018867-76018880 | A | intron | IMPG1 | LUSC, OV |
| chr4:145110085-145110100 | T | intron | ABCE1 | LUSC, SKCM |
| chr1:94498584-94498598 | T | intron | ABCD3 | LUSC, SKCM |
| chr1:52481679-52481693 | A | intron | ZCCHC11 | LUSC, SKCM |
| chr12:7368968-7368991 | GA | intron | CD163L1 | LUSC, SKCM |

Genomic coordinates that have ‘HG19’ mentioned on the side were partially deleted in HG38 hence, the coordinates from the previous build are given.

**Supplementary table 7: Out of 144 MST loci computationally found to be specific for other cancer types, 137 produced data in both sample groups.** All loci produced at least 10 reads per loci in at least 10 samples in both sample groups. The genomic coordinates furnished correspond to the HG38 genome reference build.

| **Genomic position** | **Repeat** | **Gene region** | **Gene** | **Disease** |
| --- | --- | --- | --- | --- |
| chr8:39749565-39749600 | GT | intron | ADAM2 | BC |
| chr8:23852057-23852082 | TG | intron | STC1 | BC |
| chr2:202765380-202765400 | T | intron | FAM117B | BC |
| chr3:155116591-155116607 | TA | intron | MME | BC |
| chr3:113360927-113360938 | A | intron | WDR52 | BC |
| chr16:20944777-20944802 | AC | intron | DNAH3 | BC |
| chr12:110396226-110396243 | A | intron | ANAPC7 | BC |
| chr16:56684104-56684123 | T | exon | MT1X | BC |
| chr4:76144324-76144338 | A | intron | NUP54 | BC |
| chr14:102083733-102083750 | A | intron | HSP90AA1 | BC |
| chr17:59586236-59586253 | A | intron | DHX40 | BC |
| chr20:20038239-20038260 | A | intron | CRNKL1 | BC |
| chr7:148797703-148797719 | T | intron | CUL1 | BC |
| chrX:10141619-10141634 | A | exon | WWC3 | BC |
| chr1:113829711-113829722 | A | intron | PTPN22 | BC |
| chr10:45073089-45073105 | T | intergenic | - | BC |
| chr15:81345017-81345037 | GA | intron | TMC3 | BC |
| chr4:54264835-54264851 | A | intron | PDGFRA | BC |
| chr22:37912036-37912064 | TG | intron | MICALL1 | BC |
| chr18:46812342-46812357 | A | exon | PIAS2 | BC |
| chr5:87383679-87383696 | A | intron | RASA1 | BC |
| chr3:33836009-33836020 | T | intron | PDCD6IP | BC |
| chr2:197469873-197469884 | A | intron | COQ10B | BC |
| chr2:75692147-75692171 | AT | intron | C2orf3 | BC |
| chr3:198153260-198153301 | GCA | exon | FAM157A | BC |
| chr3:196257948-196257959 | A | intron | PCYT1A | BC |
| chr11:118482323-118482338 | T | intron | MLL | BC |
| chr15:83804574-83804590 | T | intron | ADAMTSL3 | BC |
| chr1:23082431-23082446 | T | intron | AOF2 | BC |
| chrX:71592599-71592613 | T | intron | ACRC | BC, GBM |
| chr4:47744586-47744598 | A | intron | CORIN | BC, GBM, MB |
| chr8:106692713-106692726 | A | intron | OXR1 | BC, GBM, OV |
| chr2:202815832-202815844 | A | intron | ICA1L | BC, GBM, OV |
| chr7:38242530-38242549 | GT | intron | TRG | BC, MB |
| chr9:5798652-5798666 | A | intron | ERMP1 | BC, OV |
| chr17:65750900-65750913 | A | intron | CCDC46 | BC, OV |
| chr20:5186510-5186522 | T | intron | CDS2 | BC, OV |
| chr7:123117666-123117678 | A | intron | SLC13A1 | BC, OV |
| chr11:110258201-110258215 | A | intron | RDX | BC, OV |
| chr6:170572302-170572314 | T | exon | TBP | BC, OV |
| chr8:31076301-31076312 | T | intron | WRN | BC, OV |
| chr3:98580864-98580876 | A | intron | CPOX | BC, OV |
| chr11:62798437-62798472 | AAAAGA | intron | NXF1 | BC, OV |
| chr15:89268652-89268664 | T | intron | FANCI | BC, OV |
| chr5:134608354-134608369 | T | intron | SAR1B | BC, OV |
| chr19:29615224-29615240 | T | intron | POP4 | BC, OV |
| chr15:62748318-62748333 | A | intron | TLN2 | BC, SKCM |
| chr6:70240579-70240595 | AT | intron | COL9A1 | GBM |
| chr3:171126228-171126241 | A | intron | TNIK | GBM |
| chr16:70839964-70839978 | T | intron | HYDIN | GBM |
| chr4:168275913-168275928 | A | intron | DDX60 | GBM |
| chr14:95099732-95099772 | AC | intron | DICER1 | GBM |
| chr17:56904211-56904226 | A | intron | TRIM25 | GBM |
| chr4:188142208-188142243 | GT | intron | TRIML1 | GBM |
| chr7:103185541-103185553 | A | 3utr | DPY19L2P2 | GBM |
| chr7:73307734-73307743 | CAA | exon | NSUN5 | GBM |
| chr21:10516457-10516469 | A | intergenic | - | GBM |
| chr7:83392484-83392501 | A | intron | SEMA3E | GBM |
| chr10:87057822-87057837 | A | intron | GLUD1 | GBM |
| chr15:43618669-43618701 | CAG | exon | STRC | GBM |
| chr14:35865700-35865714 | T | intron | BRMS1L | GBM |
| chr10:121496816-121496831 | T | intron | FGFR2 | GBM |
| chr3:121483587-121483611 | A | intron | POLQ | GBM |
| chr2:138550814-138550849 | TC | intron | SPOPL | GBM |
| chr3:113000945-113000960 | A | exon | GTPBP8 | GBM |
| chr3:154284569-154284580 | T | intron | DHX36 | GBM |
| chr11:119274082-119274098 | T | intron | CBL | GBM |
| chr1:225519570-225519585 | A | intron | ENAH | GBM |
| chr1:117062509-117062522 | T | intron | TTF2 | GBM |
| chr12:33426063-33426109 | CA | intron | SYT10 | GBM |
| chr2:91698005-91698016 | A | intergenic | - | GBM, OV |
| chr9:52626-52640 | A | intergenic | - | GBM, OV, SKCM |
| chr14:50595519-50595543 | TC | intron | ATL1 | LGG |
| chr14:21468604-21468616 | A | intron | RAB2B | LGG |
| chr17:15613747-15613758 | A | intron | CDRT1 | LGG |
| chr7:96146537-96146550 | A | intron | SLC25A13 | LGG |
| chrX:18164978-18164992 | A | exon | BEND2 | LGG |
| chr1:145456733-145456746 (HG19) | A | intron | POLR3GL | LGG |
| chr3:132447305-132447317 | T | intron | DNAJC13 | LGG |
| chr10:100505295-100505339 | CA | intron | SEC31B | LGG, MB |
| chr19:21375214-21375230 | TG | intergenic | - | LGG, OV |
| chr16:70142419-70142432 | T | intron | PDPR | LGG, SKCM |
| chr12:50660091-50660105 | T | intron | DIP2B | LGG, SKCM |
| chr13:114236623-114236635 | T | intron | CDC16 | LGG, SKCM |
| chr12:129081740-129081756 | T | intron | TMEM132D | MB |
| chr5:36629700-36629712 | A | intron | SLC1A3 | MB |
| chr11:17089651-17089679 | GT | exon | PIK3C2A | MB |
| chr10:119037061-119037072 | C | intron | EIF3A | MB, SKCM |
| chr2:233460070-233460083 | A | intron | DGKD | OV |
| chr6:49848161-49848174 | T | intron | CRISP1 | OV |
| chr3:50057664-50057685 | T | intron | RBM6 | OV |
| chr17:68045756-68045769 | T | intron | KPNA2 | OV |
| chr17:49821919-49821932 | A | intron | MYST2 | OV |
| chr19:20646413-20646427 | AC | intron | ZNF626 | OV |
| chr14:91462502-91462516 | T | intron | SMEK1 | OV |
| chr12:106106383-106106396 | A | intron | NUAK1 | OV |
| chr13:49376888-49376921 | ATAG | intron | CAB39L | OV |
| chr4:71022616-71022630 | T | intron | DCK | OV |
| chr7:31092622-31092634 | T | intron | ADCYAP1R1 | OV |
| chr7:82066527-82066542 | A | intron | CACNA2D1 | OV |
| chr7:36425998-36426012 | T | intron | ANLN | OV |
| chrX:11169774-11169785 | T | intron | ARHGAP6 | OV |
| chr10:67939722-67939740 | AT | intron | HERC4 | OV |
| chr10:92506574-92506588 | T | intron | IDE | OV |
| chr10:22226073-22226095 | A | intergenic | - | OV |
| chr15:64680562-64680589 | TG | intron | ZNF609 | OV |
| chr1:236558153-236558165 | A | intron | HEATR1 | OV |
| chr1:149929094-149929109 | A | intron | MTMR11 | OV |
| chr10:91819355-91819375 | T | intron | TNKS2 | OV |
| chr18:23540418-23540433 | A | intron | NPC1 | OV |
| chr8:120506629-120506642 | T | intron | MTBP | OV |
| chr2:222474811-222474831 | T | intron | SGPP2 | OV |
| chr11:89800992-89801004 | A | intron | TRIM49 | OV |
| chr11:30417412-30417426 | T | intron | MPPED2 | OV |
| chr1:169586130-169586142 | A | intron | F5 | OV |
| chr5:87383860-87383873 | T | intron | RASA1 | OV |
| chr5:159084572-159084586 | A | intron | EBF1 | OV |
| chr5:123378441-123378458 | A | intron | CEP120 | OV |
| chr18:2960515-2960527 | A | intron | LPIN2 | OV |
| chr12:75508182-75508196 | A | intron | KRR1 | OV |
| chr4:140527442-140527455 | T | intron | ELMOD2 | OV, SKCM |
| chr6:88929270-88929284 | A | intron | RNGTT | OV, SKCM |
| chr8:98042609-98042620 | A | intron | RPL30 | SKCM |
| chr6:125928610-125928624 | T | intron | NCOA7 | SKCM |
| chr16:3508263-3508275 | T | intron | CLUAP1 | SKCM |
| chr20:59922427-59922441 | A | exon | SYCP2 | SKCM |
| chr7:138749322-138749335 | A | intron | ATP6V0A4 | SKCM |
| chr6:100540391-100540403 | A | intron | ASCC3 | SKCM |
| chr15:32424717-32424732 | A | 3utr | FAM7A1 | SKCM |
| chr11:115209592-115209633 | TGG | exon | CADM1 | SKCM |
| chr18:46844689-46844703 | T | intron | PIAS2 | SKCM |
| chr3:180961448-180961461 | T | intron | FXR1 | SKCM |
| chr1:243572909-243572923 | T | intron | AKT3 | SKCM |
| chr5:138177681-138177692 | A | intron | BRD8 | SKCM |
| chr19:21167718-21167729 | A | intron | ZNF431 | SKCM |
| chr19:4947051-4947063 | T | intron | UHRF1 | SKCM |
| chr12:95992869-95992882 | CCCT | intron | HAL | SKCM |

**Supplementary table 8: Out of 84 MST loci used as control, 79 produced data in both sample groups.** All loci produced at least 10 reads per loci in at least 10 samples in both sample groups. The genomic coordinates furnished attribute to the HG38 build.

A set of 84 control loci were added to the enrichment kit to demonstrate the hypermutable nature of forensic and paternity test MST loci and the resilience of random MST loci in exonic regions (highly conserved) of the genome to MSI in both the lung cancer and non-cancer control sample groups. Of the 84 control loci, 79 were reliably called in both sample groups. About 70% of the 64 control loci found in exon regions were found to have the same predominant genotype (found in greater than 50% of the control or cancer samples) in both sample groups while 86% of the 15 hyper mutable loci were found to have a wide spectrum of genotypes. None of the 79 control loci were found to have consistent genotypes that differed between the sample groups (cancer and normal), that is none of the control loci were informative for differentiating the two groups, as expected.

| **Genomic position** | **Repeat** | **Gene region** | **Gene/ Repeat ID** | |
| --- | --- | --- | --- | --- |
| chr8:19957980-19958041 | AAAT | intron | LPL | |
| chr8:25503396-25503417 | GAAAG | exon | CDCA2 | |
| chr1:209432292-209432332 | AGC | exon | LOC642587 | |
| chr2:48515217-48515259 | TC | exon | KLRAQ1 | |
| chr2:1489620-1489686 | AATG | intron | TPOX | |
| chr6:110895439-110895479 | GTTTT | exon | AMD1 | |
| chr7:149239672-149239691 | CGG | exon | ZNF212 | |
| chr6:109632842-109632919 | ATAG | exon | AKD1 | |
| chr3:49115555-49115580 | TCTTCC | exon | USP19 | |
| chr3:18349641-18349687 | CTG | exon | SATB1 | |
| chr16:69693627-69693682 | CAG | exon | NFAT5 | |
| chr12:49033867-49033913 | TGC | exon | MLL2 | |
| chr12:114355457-114355501 | TC | exon | TBX5 | |
| chr9:127506124-127506152 | CTCA | exon | FAM129B | |
| chr9:132896597-132896621 | GCT | exon | TSC1 | |
| chr9:71768574-71768616 | CCTCCG | exon | TMEM2 | |
| chr16:67980293-67980324 | GCA | exon | DPEP3 | |
| chr17:58756094-58756155 | CCGAAC | exon | PPM1E | |
| chr17:12992120-12992153 | TGAT | exon | ELAC2 | |
| chr17:50835991-50836015 | GCT | exon | WFIKKN2 | |
| chr17:17136248-17136283 | CAG | exon | MPRIP | |
| chr4:90127564-90127590 | CT | exon | FAM190A | |
| chr4:139889485-139889540 | GCT | exon | MAML3 | |
| chr4:68349770-68349795 | CCG | exon | YTHDC1 | |
| chr19:47742125-47742165 | TTCC | exon | EHD2 | |
| chr14:94842043-94842108 | [CTGT]n[CTAT]n | | D14S1434 |  |
| chr14:61321907-61321932 | GGGA | exon | PRKCH |  |
| chr14:103385444-103385464 | GCG | exon | MARK3 |  |
| chr14:22883256-22883286 | GCACAC | exon | REM2 |  |
| chr12:76031129-76031189 | GCT | exon | PHLDA1 |  |
| chr12:4914286-4914330 | ACAA | exon | KCNA1 |  |
| chr12:5983962-5984074 | AGAT | intron | VWA |  |
| chr17:42699813-42699840 | GCTGT | exon | CNTNAP1 |  |
| chr17:81996788-81996845 | AGCAGG | exon | ASPSCR1 |  |
| chr17:52158102-52158125 | GCG | exon | CA10 |  |
| chr4:54740022-54740046 | AAAAC | exon | KIT |  |
| chr4:93829017-93829037 | CCG | exon | ATOH1 |  |
| chr20:49905305-49905338 | AAAAC | exon | SPATA2 |  |
| chrX:53077999-53078047 | TC | exon | GPR173 |  |
| chrX:134481487-134481569 | AGAT | intron | HPRTB |  |
| chr1:965304-965347 | TTTA | exon | KLHL17 |  |
| chr1:15746939-15746954 | TGC | exon | TMEM82 |  |
| chr6:6321353-6321413 | AAAG |  |  |  |
| chr6:45422678-45422748 | GCA | exon | RUNX2 |  |
| chr10:129294237-129294302 | GGAA |  | D10S1248 |  |
| chr10:75028875-75028954 | GAGGAA | exon | MYST4 |  |
| chr15:40036336-40036424 | CTG | exon | SRP14 |  |
| chr1:247876957-247876991 | CT | exon | TRIM58 |  |
| chr1:2556549-2556574 | TTCTCT | exon | TNFRSF14 |  |
| chr1:26429657-26429681 | CTTCC | exon | LIN28 |  |
| chrY:18639710-18639764 | GAAA |  | DYS385_a/b |  |
| chr11:3855884-3855906 | TCTCT | exon | STIM1 |  |
| chr3:45540732-45540809 | AGAT |  | D3S1358 |  |
| chr10:117548492-117548574 | GA | exon | EMX2 |  |
| chr10:49016119-49016146 | TTTG | exon | C10orf72 |  |
| chr3:126988622-126988658 | TGC | exon | PLXNA1 |  |
| chr18:63281662-63281742 | GAAA |  | D18S51 |  |
| chr22:37140281-37140335 | ATT |  | D22S1045 |  |
| chr18:69867388-69867414 | TCTCT | exon | CD226 |  |
| chr8:144530000-144530021 | CAGGA | exon | KIAA1688 |  |
| chr5:113017812-113017852 | ATATCT | exon | DCP2 |  |
| chr5:88684765-88684804 | TC | exon | LOC645323 |  |
| chr5:153490748-153490778 | AAGG | exon | GRIA1 |  |
| chr11:2171078-2171122 | AATG | intron | TH01 |  |
| chr16:67842859-67842951 | GCA | exon | THAP11 |  |
| chr1:226993391-226993405 | TGC | exon | CDC42BPA |  |
| chr5:150076298-150076384 | AGAT | intron | CSF1PO |  |
| chr5:146233608-146233675 | CCAGGC | exon | RBM27 |  |
| chr5:148982979-148983006 | TGACAT | exon | SH3TC2 |  |
| chr5:150651587-150651611 | CTGGG | exon | SYNPO |  |
| chr19:11164698-11164760 | CCAT | exon | KANK2 |  |
| chr19:11466768-11466792 | GGGCC | exon | ELAVL3 |  |
| chr19:29926209-29926306 | (AAGG)(AAAG)(AAGG)(TAGG)[AAGG]n | | D19S433 |  |
| chr19:2514952-2515006 | GA | exon | GNG7 |  |
| chr18:37243909-37243929 | CGG | exon | BRUNOL4 |  |
| chr11:6390698-6390746 | GGCGCT | exon | SMPD1 |  |
| chr12:12297001-12297100 | [AGAT]8-17[AGAC]6-10[AGAT]0-1 | | D12S391 |  |
| chr12:114406047-114406082 | TC | exon | TBX5 |  |
| chr12:12643661-12643693 | TCTAG | exon | CREBL2 |  |

**Supplementary table 9: The leave one out cross validation confirms that the predictive power of the model sustains when applied to a test set.** The model consistently predicted 28 out of 30 lung cancer germlines samples to be ‘at-risk’ and 88 out of 89 thousand genome non-cancer control samples to be ‘healthy’. The explanations of the sample types are as follows: LC – Lung cancer, 1kGPC – 1000 genome project control.

| **Sample #** | **Sample Type** | **% of loci with cancer GT** | **Leave one out at-risk % cut off** | **Leave-one-out prediction** |
| --- | --- | --- | --- | --- |
| 1 | LC | 95.2 | 52.4 | At risk |
| 2 | LC | 63.6 | 50.0 | At risk |
| 3 | LC | 81.0 | 52.4 | At risk |
| 4 | LC | 54.5 | 50.0 | At risk |
| 5 | LC | 68.2 | 50.0 | At risk |
| 6 | LC | 57.1 | 52.4 | At risk |
| 7 | LC | 72.7 | 50.0 | At risk |
| 8 | LC | 81.8 | 50.0 | At risk |
| 9 | LC | 95.2 | 52.4 | At risk |
| 10 | LC | 77.3 | 50.0 | At risk |
| 11 | LC | 76.2 | 52.4 | At risk |
| 12 | LC | 47.6 | 52.4 | Healthy |
| 13 | LC | 57.1 | 52.4 | At risk |
| 14 | LC | 71.4 | 52.4 | At risk |
| 15 | LC | 90.5 | 52.4 | At risk |
| 16 | LC | 71.4 | 52.4 | At risk |
| 17 | LC | 72.7 | 50.0 | At risk |
| 18 | LC | 63.6 | 50.0 | At risk |
| 19 | LC | 66.7 | 52.4 | At risk |
| 20 | LC | 68.2 | 50.0 | At risk |
| 21 | LC | 72.7 | 50.0 | At risk |
| 22 | LC | 59.1 | 50.0 | At risk |
| 23 | LC | 57.1 | 52.4 | At risk |
| 24 | LC | 81.8 | 50.0 | At risk |
| 25 | LC | 31.8 | 50.0 | Healthy |
| 26 | LC | 66.7 | 52.4 | At risk |
| 27 | LC | 63.6 | 50.0 | At risk |
| 28 | LC | 71.4 | 52.4 | At risk |
| 29 | LC | 50.0 | 50.0 | At risk |
| 30 | LC | 90.5 | 52.4 | At risk |
| 31 | 1KGPC | 25.0 | 55.0 | Healthy |
| 32 | 1KGPC | 45.0 | 55.0 | Healthy |
| 33 | 1KGPC | 47.6 | 52.4 | Healthy |
| 34 | 1KGPC | 40.0 | 55.0 | Healthy |
| 35 | 1KGPC | 40.0 | 55.0 | Healthy |
| 36 | 1KGPC | 52.4 | 52.4 | Healthy |
| 37 | 1KGPC | 42.9 | 52.4 | Healthy |
| 38 | 1KGPC | 30.0 | 55.0 | Healthy |
| 39 | 1KGPC | 25.0 | 55.0 | Healthy |
| 40 | 1KGPC | 50.0 | 59.1 | Healthy |
| 41 | 1KGPC | 57.1 | 52.4 | At risk |
| 42 | 1KGPC | 47.6 | 52.4 | Healthy |
| 43 | 1KGPC | 40.0 | 55.0 | Healthy |
| 44 | 1KGPC | 38.1 | 52.4 | Healthy |
| 45 | 1KGPC | 38.1 | 52.4 | Healthy |
| 46 | 1KGPC | 25.0 | 55.0 | Healthy |
| 47 | 1KGPC | 47.6 | 52.4 | Healthy |
| 48 | 1KGPC | 47.6 | 52.4 | Healthy |
| 49 | 1KGPC | 31.8 | 59.1 | Healthy |
| 50 | 1KGPC | 40.0 | 55.0 | Healthy |
| 51 | 1KGPC | 33.3 | 52.4 | Healthy |
| 52 | 1KGPC | 52.4 | 52.4 | Healthy |
| 53 | 1KGPC | 52.4 | 52.4 | Healthy |
| 54 | 1KGPC | 40.0 | 55.0 | Healthy |
| 55 | 1KGPC | 38.1 | 52.4 | Healthy |
| 56 | 1KGPC | 35.0 | 55.0 | Healthy |
| 57 | 1KGPC | 52.4 | 52.4 | Healthy |
| 58 | 1KGPC | 38.1 | 52.4 | Healthy |
| 59 | 1KGPC | 45.5 | 59.1 | Healthy |
| 60 | 1KGPC | 33.3 | 52.4 | Healthy |
| 61 | 1KGPC | 45.5 | 59.1 | Healthy |
| 62 | 1KGPC | 33.3 | 52.4 | Healthy |
| 63 | 1KGPC | 38.1 | 52.4 | Healthy |
| 64 | 1KGPC | 14.3 | 52.4 | Healthy |
| 65 | 1KGPC | 31.8 | 59.1 | Healthy |
| 66 | 1KGPC | 28.6 | 52.4 | Healthy |
| 67 | 1KGPC | 50.0 | 59.1 | Healthy |
| 68 | 1KGPC | 42.9 | 52.4 | Healthy |
| 69 | 1KGPC | 25.0 | 55.0 | Healthy |
| 70 | 1KGPC | 36.4 | 59.1 | Healthy |
| 71 | 1KGPC | 50.0 | 55.0 | Healthy |
| 72 | 1KGPC | 40.0 | 55.0 | Healthy |
| 73 | 1KGPC | 40.9 | 59.1 | Healthy |
| 74 | 1KGPC | 38.1 | 52.4 | Healthy |
| 75 | 1KGPC | 45.5 | 59.1 | Healthy |
| 76 | 1KGPC | 18.2 | 59.1 | Healthy |
| 77 | 1KGPC | 25.0 | 55.0 | Healthy |
| 78 | 1KGPC | 33.3 | 52.4 | Healthy |
| 79 | 1KGPC | 42.9 | 52.4 | Healthy |
| 80 | 1KGPC | 9.5 | 52.4 | Healthy |
| 81 | 1KGPC | 36.4 | 59.1 | Healthy |
| 82 | 1KGPC | 23.8 | 52.4 | Healthy |
| 83 | 1KGPC | 23.8 | 52.4 | Healthy |
| 84 | 1KGPC | 35.0 | 55.0 | Healthy |
| 85 | 1KGPC | 28.6 | 52.4 | Healthy |
| 86 | 1KGPC | 47.6 | 52.4 | Healthy |
| 87 | 1KGPC | 36.4 | 59.1 | Healthy |
| 88 | 1KGPC | 36.4 | 59.1 | Healthy |
| 89 | 1KGPC | 20.0 | 55.0 | Healthy |
| 90 | 1KGPC | 23.8 | 52.4 | Healthy |
| 91 | 1KGPC | 45.5 | 59.1 | Healthy |
| 92 | 1KGPC | 40.0 | 55.0 | Healthy |
| 93 | 1KGPC | 42.9 | 52.4 | Healthy |
| 94 | 1KGPC | 50.0 | 59.1 | Healthy |
| 95 | 1KGPC | 50.0 | 59.1 | Healthy |
| 96 | 1KGPC | 42.9 | 52.4 | Healthy |
| 97 | 1KGPC | 31.8 | 59.1 | Healthy |
| 98 | 1KGPC | 25.0 | 55.0 | Healthy |
| 99 | 1KGPC | 40.0 | 55.0 | Healthy |
| 100 | 1KGPC | 50.0 | 59.1 | Healthy |
| 101 | 1KGPC | 38.1 | 52.4 | Healthy |
| 102 | 1KGPC | 35.0 | 55.0 | Healthy |
| 103 | 1KGPC | 15.0 | 55.0 | Healthy |
| 104 | 1KGPC | 40.0 | 55.0 | Healthy |
| 105 | 1KGPC | 31.8 | 59.1 | Healthy |
| 106 | 1KGPC | 10.0 | 55.0 | Healthy |
| 107 | 1KGPC | 25.0 | 55.0 | Healthy |
| 108 | 1KGPC | 54.5 | 59.1 | Healthy |
| 109 | 1KGPC | 23.8 | 52.4 | Healthy |
| 110 | 1KGPC | 40.9 | 59.1 | Healthy |
| 111 | 1KGPC | 20.0 | 55.0 | Healthy |
| 112 | 1KGPC | 42.9 | 52.4 | Healthy |
| 113 | 1KGPC | 27.3 | 59.1 | Healthy |
| 114 | 1KGPC | 33.3 | 52.4 | Healthy |
| 115 | 1KGPC | 27.3 | 59.1 | Healthy |
| 116 | 1KGPC | 47.6 | 52.4 | Healthy |
| 117 | 1KGPC | 36.4 | 59.1 | Healthy |
| 118 | 1KGPC | 42.9 | 52.4 | Healthy |
| 119 | 1KGPC | 45.5 | 59.1 | Healthy |

Detailed methods: The cross-validation leave-one-out procedure entails: 1. the extraction of a list of markers that can consistently differentiate between the cancer and the control samples, 2. building the binary classifier based on the extracted MSTs list and 3. calculating the sensitivity and specificity of the classifier.

This procedure was repeated 119 times (30 cancer + 89 control). Every iteration, one sample is left out. The MST list is produced based on the consistently differing predominant genotypes in the other 118 samples. The original list of MSTs is 21 while the 119 iterations produced lists with 20(26), 21(53) and 22(40) MSTs. According to the 119 iterations, on average, 70% of the list MSTs in the cancer samples have cancer genotype while only 40% of the list MSTs in the control samples have cancer genotype. Please see supplementary table 9 for detailed information.

**Supplementary table 10: Investigation of TCGA lung cancer data reveal that alterations in genes in lung cancer risk classifiers occur on average in 37% of studied individuals.**

| **TCGA lung cancer study** | **% of cases altered** |
| --- | --- |
| Lung Adenocarcinoma (TCGA, Nature 2014) | 31.7% of 230 cases |
| Lung Adenocarcinoma (TCGA, Provisional) | 33.5% of 230 cases |
| Lung Squamous Cell Carcinoma (TCGA, Nature 2012) | 39.9% of 178 cases |
| Lung Squamous Cell Carcinoma (TCGA, Provisional) | 49.7% of 177 cases |
| Pan-Lung Cancer (TCGA, Nat Genet 2016) | 34.5% of 1144 cases |

**Supplementary table 11: Analysis of TCGA Pan Cancer lung cancer studies reveal indicate that 9 gene pairs tend to significantly co-occur within lung cancer risk classifiers.**

| **Gene A** | **Gene B** | **P-value** | **Log Odds** |
| --- | --- | --- | --- |
| ATG3 | ARL13B | 0.001 | 2.82 |
| PPP1R21 | REL | 0.001 | 2.42 |
| PPP1R21 | CCDC88A | 0.001 | 2.26 |
| REL | CCDC88A | 0.001 | 3.00 |
| ATG3 | REL | 0.002 | 1.61 |
| FUBP3 | CNOT4 | 0.005 | 2.40 |
| REL | PRPF18 | 0.017 | 1.89 |
| ARID1B | IMPG1 | 0.017 | 1.02 |
| FUBP3 | ARID1B | 0.049 | 1.46 |

**Supplementary table 12: Analysis of ontologies indicates alternative splicing, acetylation, and splice variants to be significant terms among the 12 genes in lung cancer risk classifier.**

| **Term** | **Count** | **%** | **P-value** | **Genes** |
| --- | --- | --- | --- | --- |
| Alternative splicing | 12 | 92.308 | 0.005 | HSPH1, FUBP3, CCDC88A, FAM49B, REL, IMPG1, PPP1R21, ARID1B, PRPF18, ATG3, CNOT4, ARL13B |
| Acetylation | 6 | 46.154 | 0.040 | HSPH1, FUBP3, REL, ARID1B, PRPF18, ATG3 |
| Splice variant | 9 | 69.231 | 0.047 | HSPH1, FUBP3, CCDC88A, PPP1R21, ARID1B, PRPF18, ATG3, CNOT4, ARL13B |
| Coiled coil | 5 | 38.462 | 0.063 | HSPH1, CCDC88A, PPP1R21, CNOT4, ARL13B |
| Cilium | 2 | 15.385 | 0.069 | FAM49B, ARL13B |
| Ubl conjugation | 4 | 30.769 | 0.072 | FUBP3, ATG3, CNOT4, ARL13B |

**Supplementary table 13: The following table contains the gender, ethnicity and smoking status of TCGA samples used for the computational identification of LUAD and LUSC specific MST loci.** The data presented in this table represents the general distribution of non-small cell lung cancer samples in the TCGA. Both the LUAD and LUSC samples are predominantly of Caucasian descent which is matched with the 1000 genome control samples that are mostly of northern European descent. All 1000 genome control samples are from healthy individuals and hence are non-smokers. Out of the 390 non-cancer controls (1kGP), 249 are females and 141 are males.

| **GENDER** | **Male %** | **Female %** |  | **Unknown %** | **Total** |
| --- | --- | --- | --- | --- | --- |
| **LUAD** | 46 | 54 |  | 0 | 266 |
| **LUSC** | 75 | 25 |  | 0 | 222 |
| **ETHNICITY** | **Caucasian %** | **B or AF %** | **Asian %** | **Unknown %** | **Total** |
| **LUAD** | 78 | 6 | 1 | 15 | 266 |
| **LUSC** | 72 | 4 | 2 | 23 | 222 |
| **SMOKING** | **Smoker %** | **Never smoker %** | | **Unknown %** | **Total** |
| **LUAD** | 83 | 14 |  | 3 | 266 |
| **LUSC** | 94 | 4 |  | 2 | 222 |

**B – Black; AF – African American.**

**Supplementary figure 1: Callable loci distribution in the lung cancer and non-tumor samples.** In 93% of non-tumor samples and 100% of the lung cancer samples, 90 to 100% of the 326 MST loci were called. The figure shows that in the 89 + 30 target enriched samples a very high percentage of the loci included in the enrichment kit can be called with confidence.

**Supplementary figure 2: Read depth distribution in the lung cancer and non-tumor validation samples.**

**Supplementary figure 3: The 96 computationally found LUAD specific MST loci set differentiated LUAD germline samples from non-cancer 1kGP control samples with a sensitivity of 0.87 and a specificity of 0.87.** The AUC value of this prediction was found to be 0.94.


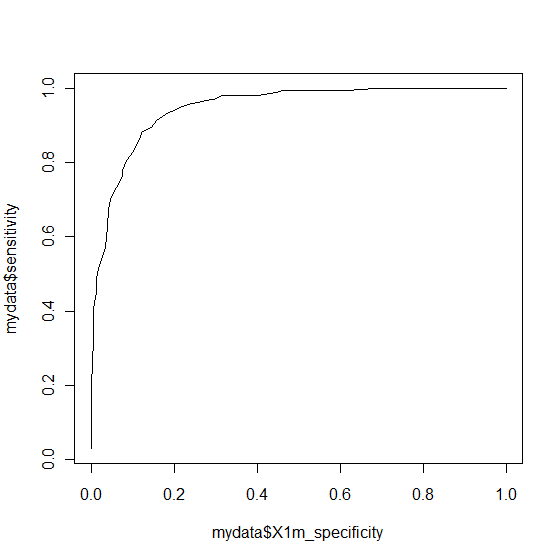


**Supplementary figure 4: The 67 computationally found LUSC specific MST loci set differentiated LUSC germline samples from non-cancer 1kGP control samples with a sensitivity of 0.88 and a specificity of 0.82.** The AUC value of this prediction was found to be 0.92.


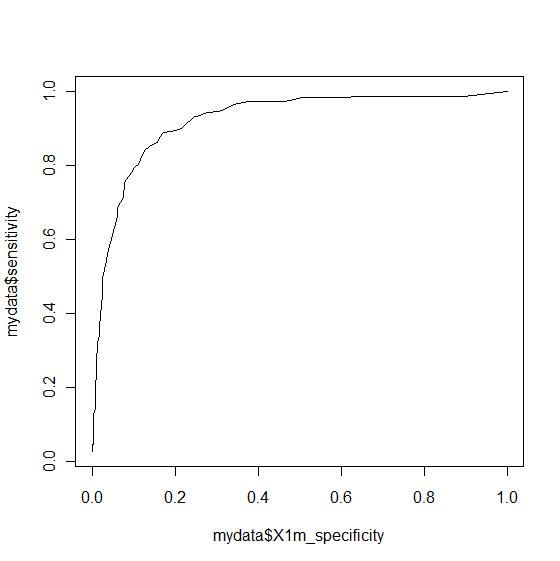


**Supplementary figure 5:** **A set of 13 MST loci that were computationally associated with lung cancer was validated by the high-depth enabled high accuracy genotyping to differentiate lung cancer samples from non-cancer 1kGP control samples with a sensitivity and specificity of 0.90 and 0.94, respectively.** (A) The AUC value for the classifier was found to be 0.96. (B) The accuracy versus cutoff plot gives the optimum point where the true positive value is high and the false positive value is low**.** According to the accuracy versus cutoff plot, a sample with 61% or more of the 13 MST loci set with predominant cancer genotype will be classified as ‘at-risk’ for NSCLC.


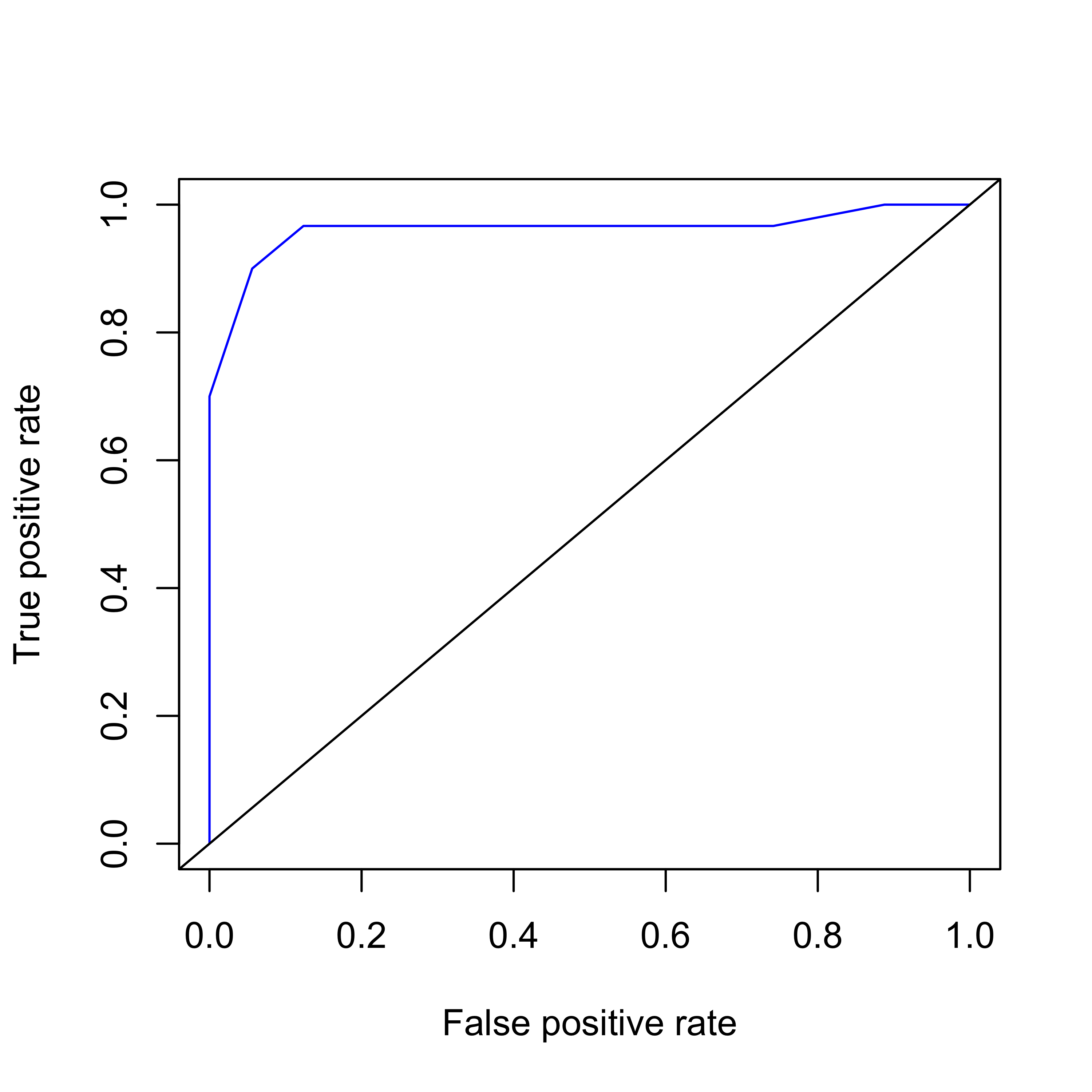

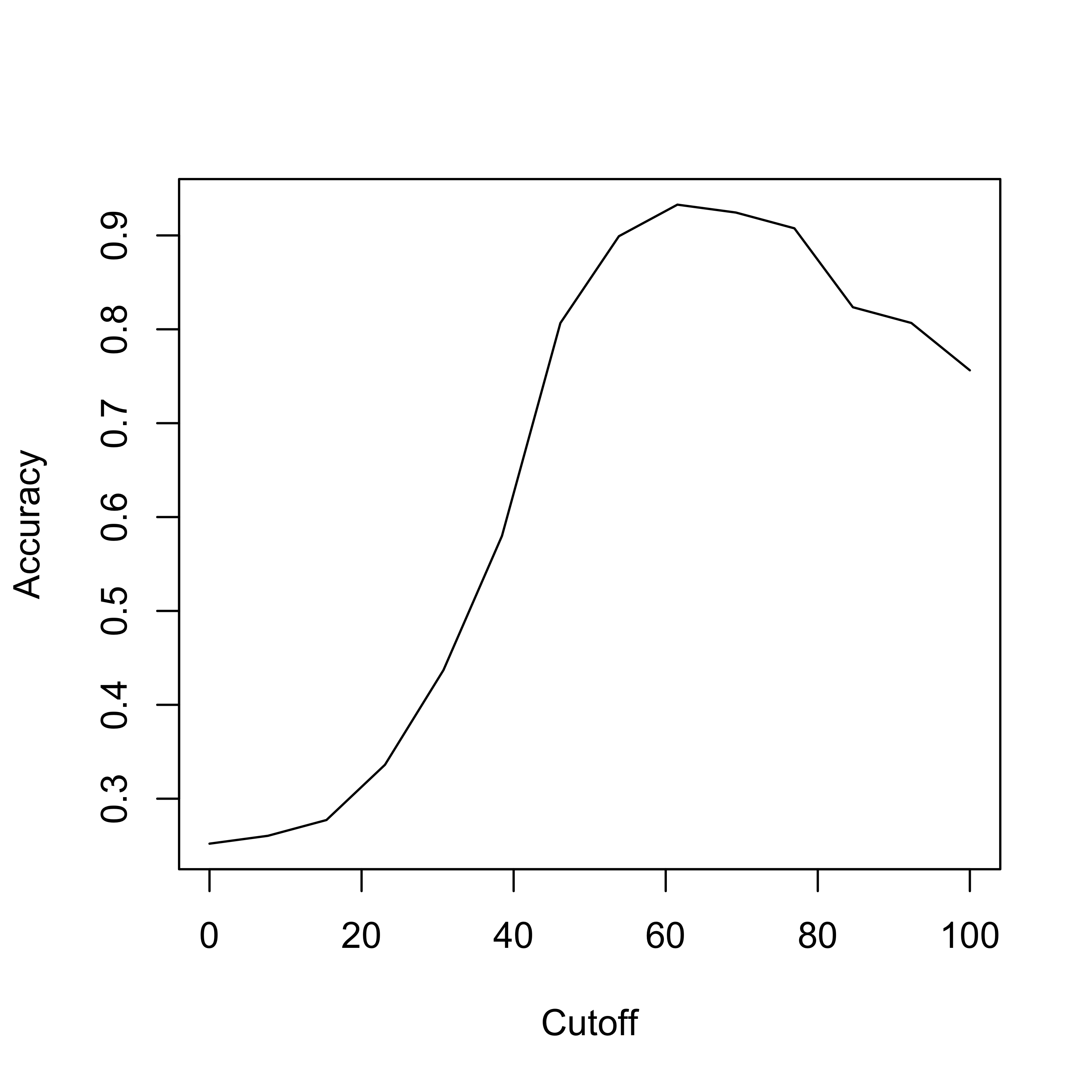


**A**

**B**

**Supplementary figure 6:** **A set of 21 (13 computationally known to be associated with lung cancer and 8 that were found to be associated for other diseases) MST loci was validated by the high-depth enabled high accuracy genotyping to differentiate lung cancer samples from non-cancer 1kGP control samples with a sensitivity and specificity of 0.93 and 0.97, respectively.** (A) The AUC value for the classifier was found to be 0.97. (B) The accuracy versus cutoff plot gives the optimum point where the true positive value is high and the false positive value is low**.** According to the accuracy versus cutoff plot, a sample with 57% or more of the 21 MST loci set with predominant cancer genotype will be classified as ‘at-risk’ for NSCLC.


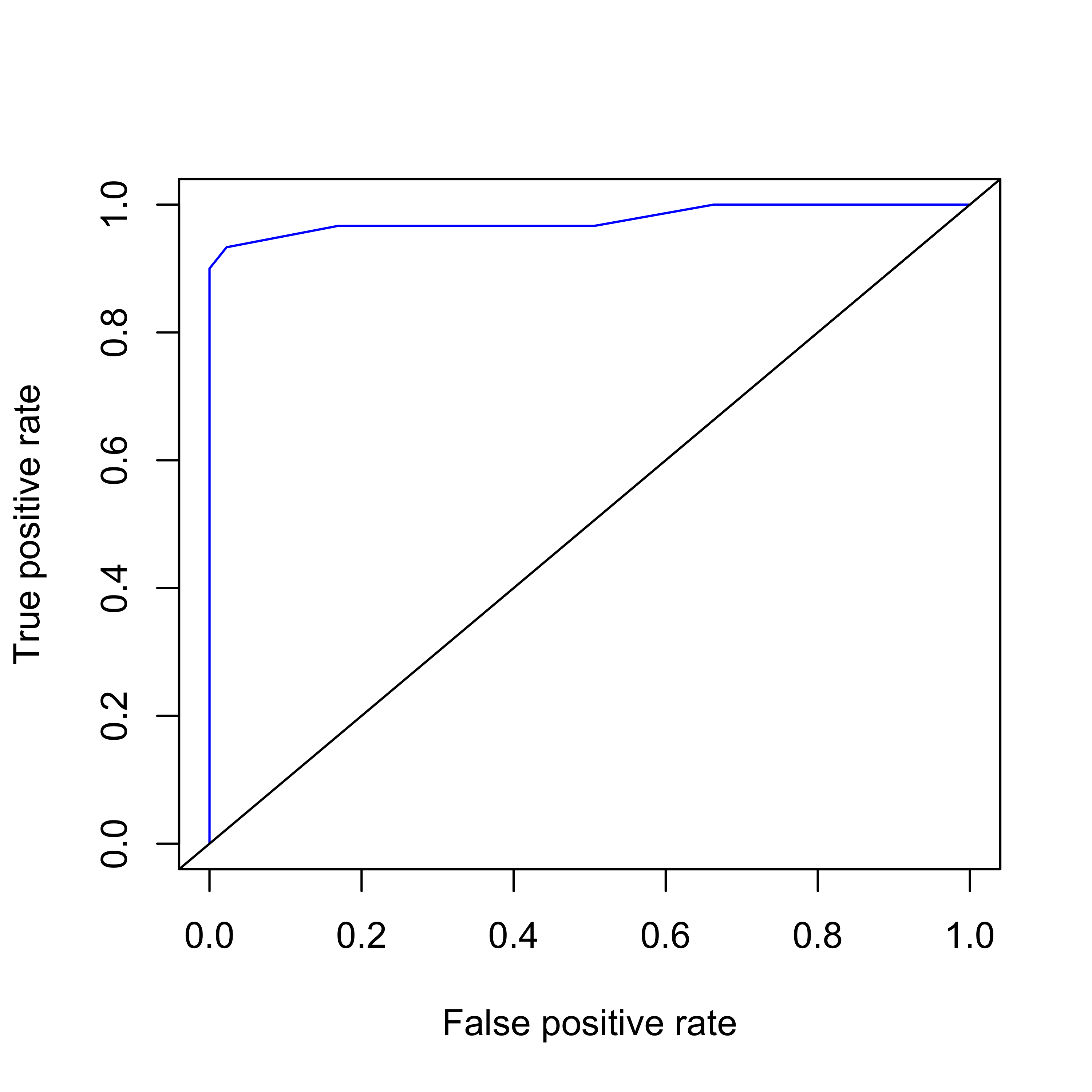

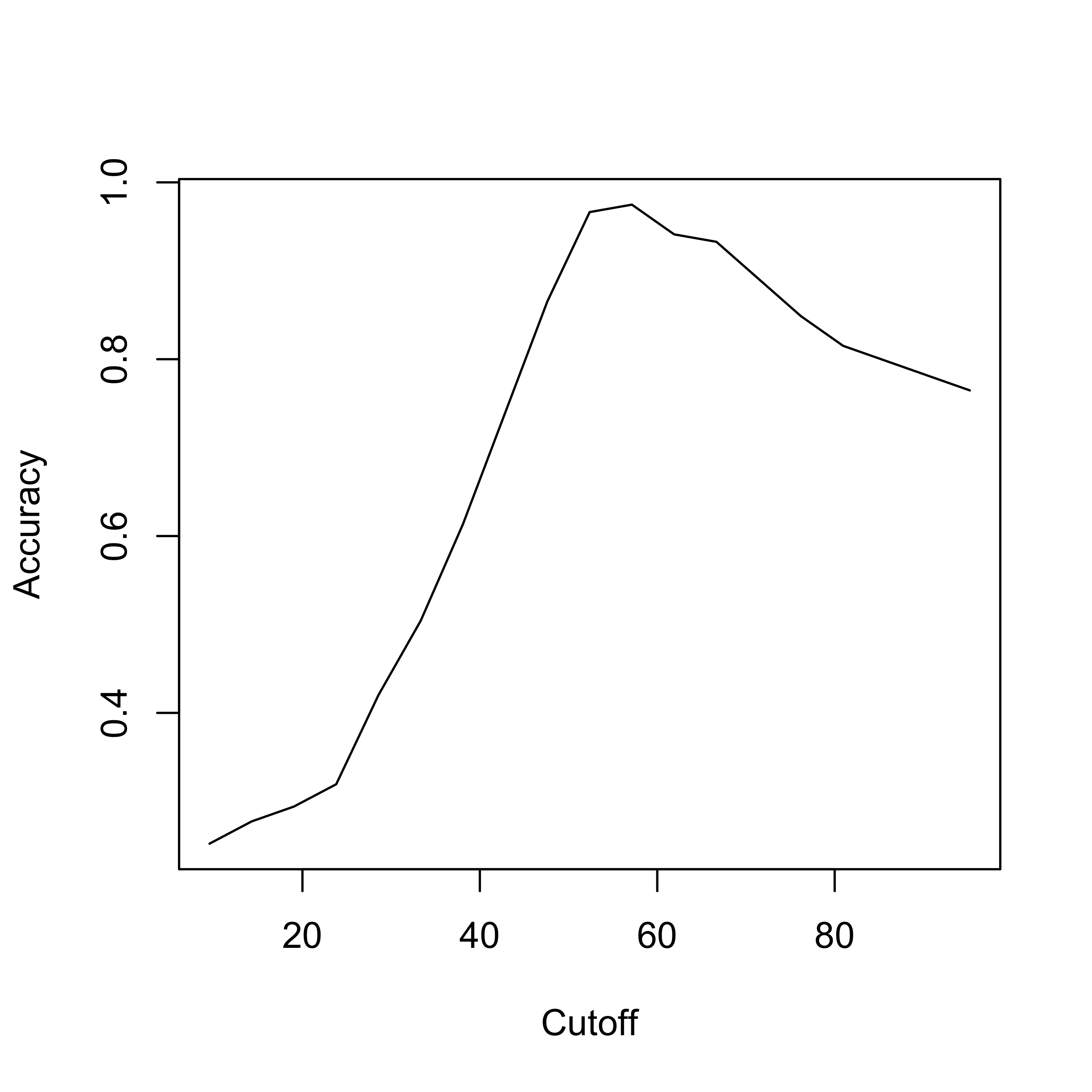


**A**

**B**

**Supplementary figure 7: Age distribution of 266 LUAD and 222 LUSC samples from the TCGA utilized for the computational identification of LUAD and LUSC specific MST loci.** The word ‘age’ represents the age at which pathological diagnosis was done. Approximately, 90% of samples from both groups fall within the age range of 50 to 80. The age information for the 1000 genomes control samples were not available but all 1000 genome sample contributors are adults, older than 18 years.


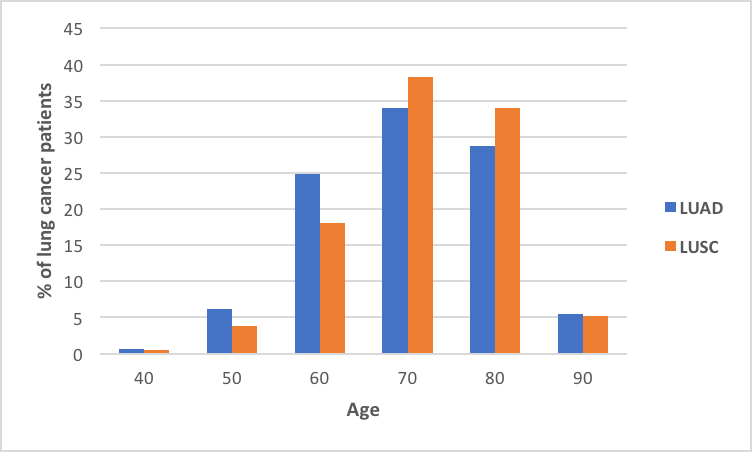


**Supplementary figure 8: The 119 iterations that were performed for the leave one out cross validation produced consistent classification results.** The following clustered-column graph shows the frequency of leave-one-out cutoff of lung cancer samples (blue - result of 30 iterations) vs. control samples (orange - result of 89 iterations). The % of list MSTs with cancer genotype in the lung cancer samples peaks between 70% and 80% while the same for the control samples peak at 40%. This convincingly shows that the leave one out analysis adequately supplements the lack of an independent validation dataset and that the classifier can be consistently extended for similar validation.


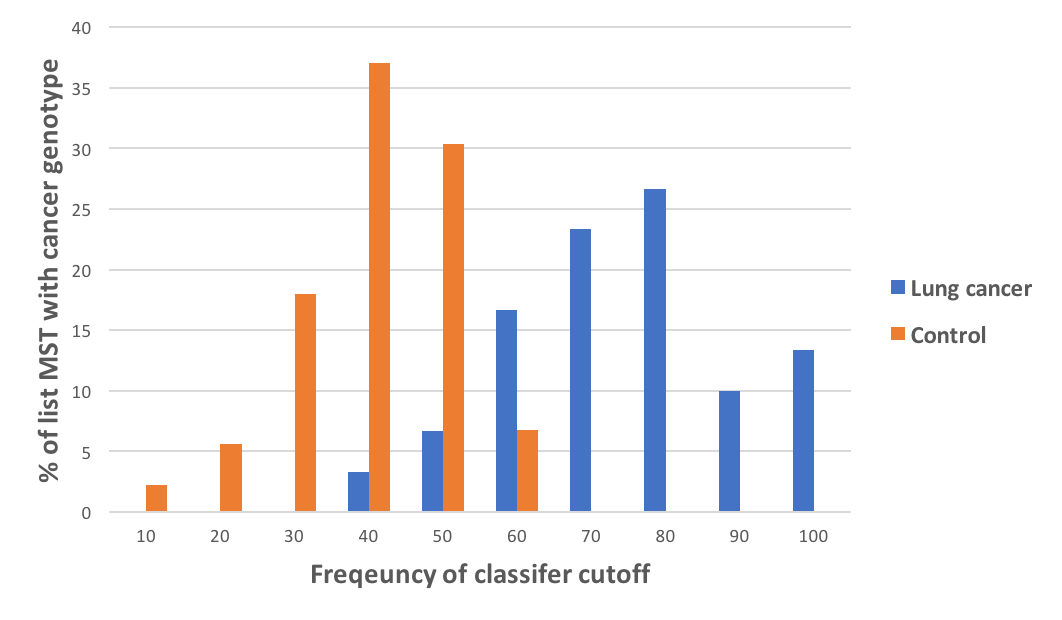


**Reference:**

1 Fonville NC, Vaksman Z, McIver LJ, Garner HR. Population analysis of microsatellite genotypes reveals a signature associated with ovarian cancer. Oncotarget 2015; 6: 11407-11420.

2 Karunasena E, McIver LJ, Rood BR, Wu X, Zhu H, Bavarva JH *et al*. Somatic intronic microsatellite loci differentiate glioblastoma from lower-grade gliomas. Oncotarget 2014; 5: 6003-6014.

3 McIver LJ, Fonville NC, Karunasena E, Garner HR. Microsatellite genotyping reveals a signature in breast cancer exomes. Breast cancer research and treatment 2014; 145: 791-798.
